# Human amnion cells reverse acute and chronic pulmonary damage in experimental neonatal lung injury

**DOI:** 10.1186/s13287-017-0689-9

**Published:** 2017-11-10

**Authors:** Dandan Zhu, Jean Tan, Amina S. Maleken, Ruth Muljadi, Siow T. Chan, Sin N. Lau, Kirstin Elgass, Bryan Leaw, Joanne Mockler, Daniel Chambers, Kristen T. Leeman, Carla F. Kim, Euan M. Wallace, Rebecca Lim

**Affiliations:** 1 The Ritchie Centre, Hudson Institute of Medical Research, Clayton, VIC Australia; 2 Department of Obstetrics and Gynaecology, Monash University, 27-31 Wright Street, Clayton, VIC 3800 Australia; 3 Monash Micro Imaging, Monash University, Clayton, Victoria Australia; 4 Queensland Lung Transplant Service, The Prince Charles Hospital, Brisbane, QLD Australia; 5 School of Medicine, The University of Queensland, Brisbane, QLD Australia; 6 Division of Newborn Medicine, Department of Paediatrics, Boston Children’s Hospital Boston, Harvard Medical School, Clayton, Victoria Australia; 7 Boston Children’s Hospital Boston Stem Cell Program, Department of Genetics, Harvard Medical School and Harvard Stem Cell Institute, Clayton, Victoria Australia

**Keywords:** Chronic neonatal lung disease, Hyperoxia, Secondary pulmonary hypertension, Intra-amniotic inflammation, Cell therapy

## Abstract

**Background:**

Despite advances in neonatal care, bronchopulmonary dysplasia (BPD) remains a significant contributor to infant mortality and morbidity. While human amnion epithelial cells (hAECs) have shown promise in small and large animal models of BPD, there is scarce information on long-term benefit and clinically relevant questions surrounding administration strategy remain unanswered. In assessing the therapeutic potential of hAECs, we investigated the impact of cell dosage, administration routes and timing of treatment in a pre-clinical model of BPD.

**Methods:**

Lipopolysaccharide was introduced intra-amniotically at day 16 of pregnancy prior to exposure to 65% oxygen (hyperoxia) at birth. hAECs were administered either 12 hours (early) or 4 days (late) after hyperoxia commenced. Collective lung tissues were subjected to histological analysis, multikine ELISA for inflammatory cytokines, FACS for immune cell populations and 3D lung stem cell culture at neonatal stage (postnatal day 7 and 14). Invasive lung function test and echocardiography were applied at 6 and 10 weeks of age.

**Results:**

hAECs improved the tissue-to-airspace ratio and septal crest density in a dose-dependent manner, regardless of administration route. Early administration of hAECs, coinciding with the commencement of postnatal hyperoxia, was associated with reduced macrophages, dendritic cells and natural killer cells. This was not the case if hAECs were administered when lung injury was established. Fittingly, early hAEC treatment was more efficacious in reducing interleukin-1β, tumour necrosis factor alpha and monocyte chemoattractant protein-1 levels. Early hAEC treatment was also associated with reduced airway hyper-responsiveness and normalisation of pressure–volume loops. Pulmonary hypertension and right ventricle hypertrophy were also prevented in the early hAEC treatment group, and this persisted until 10 weeks of age.

**Conclusions:**

Early hAEC treatment appears to be advantageous over late treatment. There was no difference in efficacy between intravenous and intratracheal administration. The benefits of hAEC administration resulted in long-term improvements in cardiorespiratory function.

**Electronic supplementary material:**

The online version of this article (doi:10.1186/s13287-017-0689-9) contains supplementary material, which is available to authorized users.

## Background

Improvements in perinatal care and clinical management have led to a decline in perinatal mortality [1]. However, the incidences of diseases of prematurity remain a significant global health burden. Bronchopulmonary dysplasia (BPD) accounts for a majority of complications associated with preterm birth, with reported incidence rates as high as 42% in infants weighing between 500 and 750 g at birth [2]. The development of BPD is multifactorial and underlying contributing factors include prenatal factors such as intrauterine inflammation, vascular maldevelopment and nutritional problems; as well as postnatal factors such as surfactant deficiency, ventilation and infection. Despite significant advances in the care of extremely premature infants over recent decades, chronic lung disease of the newborn, or BPD, remains a major cause of morbidity and mortality. It is a condition of arrested lung development resulting in the dual pathology of fewer, larger simplified alveoli and dysregulated vascular development. These culminate in impaired alveolar and vascular growth, airway injury, inflammation and fibrosis with resultant reduced lung surface area and disordered vasculature that is distant from gas exchange [3].

Currently there is no cure for established BPD. Of those infants who develop BPD, more than 10% will not survive [4] while survivors of BPD remain at risk of developing obstructive respiratory disease in adolescence and early adulthood [5]. Therapies that can effectively target the complex pathophysiology of the disease are sorely needed. Cell therapy is being explored as one approach to the treatment of BPD. Pre-clinical studies have shown that two types of stem cell-like cells may hold some promise, namely mesenchymal stem/stromal cells (MSCs) and human amnion epithelial cells (hAECs). These cells can reduce inflammation, improve alveolar architecture and promote angiogenesis in BPD-like lung injury models [6–9], and are non-tumorigenic and exhibit low antigenicity [10–12], which is suitable for allogeneic transplantation. Phase I/II clinical trials have already commenced for umbilical cord-derived MSCs (PNEUMOSTEM®) (NCT01297205, NCT01828957, NCT02381366) and hAECs (ACTRN12614000174684). Unlike the MSC trials, whose recruitment criteria included preterm infants that had not yet fulfilled the NIH criteria for BPD, the current hAEC clinical trial is recruiting only preterm infants with established BPD. Babies recruited to the hAEC trial are born earlier than 28 weeks and intubated at the point of assessment, which is conducted > 36 weeks postmenstrual age. The comparative advantage of hAECs over MSCs is that their vast cell yield from term amniotic membranes circumvents the need for expansion ex vivo [13]. They can be quickly cryopreserved and retrieved for use in critically ill babies who require rapid clinical intervention. It was to this end that we sought to determine dose effects, time-of-dose effects and the effect of route of administration on the efficacy of hAECs in the setting of BPD.

In this study, we utilised a murine neonatal lung injury model that combines antenatal inflammation with chronic postnatal hyperoxia exposure to capture several key aspects of clinical BPD. These include alveolar simplification, chronic lung inflammation and long-term sequelae including compromised respiratory function and right heart ventricular hypertrophy. We examined the dose effects of hAECs and compared the timing and routes of administration, on short-term and long-term outcomes associated with the BPD-like lung injury. Specifically, we showed that hAECs repaired BPD-like lung injury by improving the lung tissue-to-air space ratio and secondary septal crest density. These findings were concurrent with a decrease in pulmonary inflammation where tissue lysate levels of IL-1β, TNF-α, MCP-1, leukemia inhibitory factor (LIF) and macrophage inflammatory protein (MIP)-2 were significantly lowered. Further, hAEC administration was associated with a reduced pulmonary infiltration of natural killer cells, dendritic cells and macrophages. This reduction in inflammation was coincident with improved alveologenesis, as well as an increase in pulmonary endothelial cells, indicative of improved angiogenesis. Increased activation of the bronchioalveolar stem cell (BASC) niche was also observed. We also observed that hAECs rescued lung injury in a dose-dependent manner, where equal efficacy could be achieved through either intratracheal or intravenous delivery. Additionally, both early (12 hours following hyperoxia commencement, coinciding with the early stages of pulmonary inflammation) and late (4 days following hyperoxia commencement when lung injury is well underway) hAEC treatments could resolve lung injury. However, earlier cell delivery was more effective than late treatment. Intriguingly, a single dose of hAECs delivered during the neonatal period appeared to have protracted beneficial effects despite ongoing chronic hyperoxic insult. These beneficial effects persisted through adolescence and early adulthood where reduced pulmonary hypertension and right ventricle wall hypertrophy were evident. Overall, the results from this study will help inform the design of future trials using hAECs, including the design of long-term follow-up studies, thereby expediting clinical translation.

## Methods

### hAEC isolation

hAECs were isolated from amnions collected from women with a healthy pregnancy undergoing elective caesarean section at term. The mean gestational age of donors was 38 weeks ± 4 days. hAECs were isolated as described previously [14]. Cell viability was tested by Trypan blue exclusion (> 85% viability was required). hAECs isolated from individual donors were cryopreserved. hAECs isolated from the same patient were used for individual experiments.

### Animals and experimental groups

#### Short-term studies

C57/BL6 mice were time-mated and on day 16 of pregnancy (E16) injected with either 0.1 μg lipopolysaccharide (LPS from *Escherichia coli*; Sigma, St. Louis, MO, USA) in 5 μl saline or an equal volume of vehicle into each amniotic sac. Pups were then allowed to deliver naturally at term (E21). Within 12 hours of birth or on postnatal day 3.5 (PND3.5), the pups were exposed to either normoxia (FiO_2_ = 0.2) or hyperoxia (FiO_2_ = 0.65) for 7, 14 or 28 days. To prevent maternal oxygen toxicity, nursing dams were rotated every 2 days. Pregnant female mice and pups were randomly assigned to experimental groups.

### Determining the optimal dosage

**Fig. 1:**
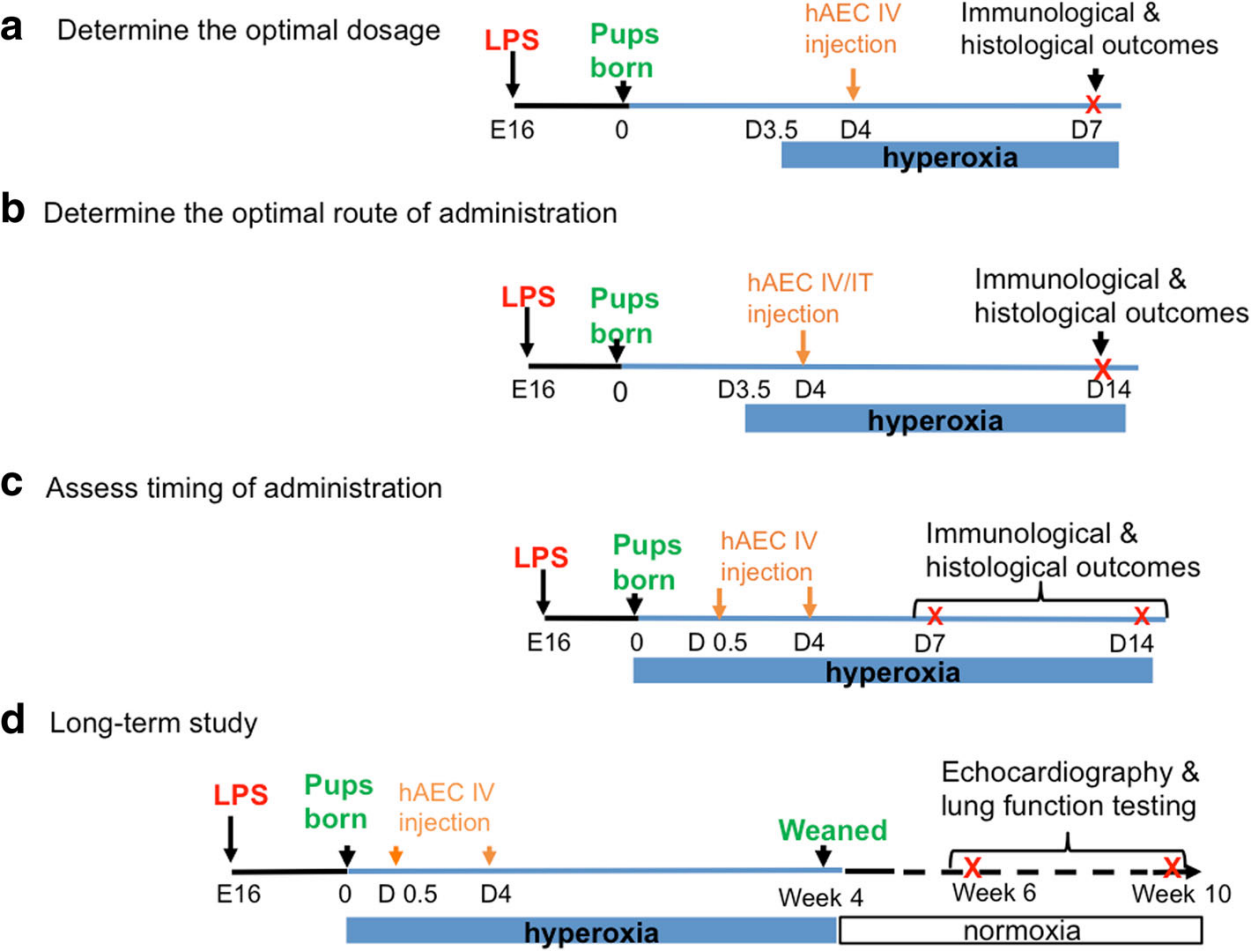
Flowchart of experimental mouse BPD model. C57/BL6 mice were time-mated and on day 16 of pregnancy (E16) injected with either 0.1 μg lipopolysaccharide (LPS) in 5 μl saline or an equal volume of vehicle into each amniotic sac. Pups were then allowed to deliver naturally at term. **a** Determine the optimal dosage. Pups were placed either in normoxia or hyperoxia chambers on PND3.5 (12 hours before hAEC treatment). On PND4, either hAECs (50,000, 75,000 or 100,000 cells) or saline were injected intravenously through the superficial temporal vein using the automated microinjection system as per the intra-amniotic injections. Mouse pups were culled on PND7. **b** Determine the optimal route of administration. Pups were exposed to either normoxia or hyperoxia from PND3.5, then the optimal dose of hAECs were injected intratracheally on PND4 and compared to the intravenous injection groups in the dose-effect experiment. The pups were culled on PND14. **c** Assess timing of administration. Pups were exposed to either normoxia or hyperoxia on PND0, and intravenous administration of 100,000 hAECs was given either on PND0.5 or on PND4. Pups were culled either on PND7 or PND14. **d** Long-term study. Pups were given hAECs intravenously either on PND0.5 or PND4, and were kept in the hyperoxia chamber from PND0 to PND28, when they were weaned and transferred to standard housing facilities. Animals were culled at either 6 or 10 weeks of age. hAEC human amnion epithelial cell, IT intratracheal, IV intravenous

The pups were placed either in normoxia or hyperoxia chambers on PND3.5 (12 hours before hAEC treatment). On PND4, either hAECs or saline were injected intravenously through the superficial temporal vein using the automated microinjection system as per the intra-amniotic injections. Mouse pups were culled on PND7 (*n* = 6–8 per group). A representation of the experimental design is shown in Fig. 1a.

The experimental groups were: (a) intra-amniotic saline + normoxia + intravenous saline; (b) intra-amniotic saline + normoxia + intravenous hAECs (100,000 cells); (c) intra-amniotic LPS + hyperoxia + intravenous saline; and (d–f) intra-amniotic LPS + hyperoxia + intravenous hAECs (50,000, 75,000 or 100,000 cells respectively).

### Determining the optimal route of administration

Pups were exposed to either normoxia or hyperoxia from PND3.5, then the optimal dose of hAECs was injected intratracheally on PND4 and compared to the intravenous injection groups in the dose-effect experiment. The pups were culled on PND14 (*n* = 6–8 per group). A representation of the experimental design is shown in Fig. 1b. These experimental groups were: (a) intra-amniotic saline + normoxia + intratracheal saline; (b) intra-amniotic saline + normoxia + intratracheal hAECs; (c) intra-amniotic LPS + hyperoxia + intratracheal saline; and (d) intra-amniotic LPS + hyperoxia + intratracheal hAECs.

### Assessing timing of administration

**Fig. 2:**
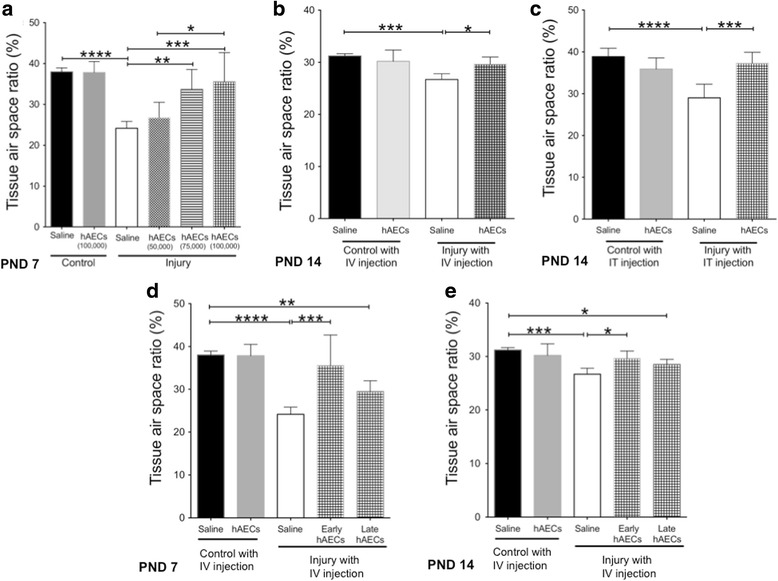
Tissue-to-air space ratio on postnatal day (PND) 7 and PND14 (*n* = 6–8). **a** Dose effect of hAEC administration. At PND7, tissue-to-air space ratio was decreased in the saline-treated injured group, but this was mitigated by the two highest doses of hAECs in a dose-dependent manner. **b** Early intravenous injection of hAECs restored the tissue-to-air space ratio on PND14. **c** Early intratracheal injection of hAECs restored the tissue-to-air space ratio on PND14. **d**, **e** Early intravenous hAEC administration normalised the tissue-to-air space ratio; however, late intravenous hAEC administration also improved lung structure but remained lower than control on both PND7 (**d**) and PND14 (**e**). Data expressed as mean ± standard error of mean (SEM). Statistical significance determined with one-way ANOVA accompanied by the Bonferroni post-hoc test. *p < 0.05, **p < 0.01, ***p < 0.001, ****p < 0.0001. hAEC human amnion epithelial cell, IT intratracheal, IV intravenous

Pups were exposed to hyperoxia on PND0, instead of PND3.5. hAEC injections were performed on either PND0.5 or PND4. We injected hAECs on PND4 for administration dosage and route optimisations because this is the only time point at which both intravenous and intratracheal routes are accessible. At PND4 there is still insufficient facial pigmentation to obscure visual identification of the superficial temporal vein and mouse pups at this age are also susceptible to the anaesthetic effects of isoflorane. To this end, we commenced hyperoxia on PND3.5 to assess ‘early’ hAEC administration for dosage and administration route optimisation—accounting for the half-day exposure to hyperoxia. When intravenous injections were used for the subsequent studies, we varied the time of cell administration instead of hyperoxia commencement. Although the mouse pups were exposed to normoxia for the first 3.5 days prior to hyperoxia commencement on PND3.5, mouse lung development at the terminal sac stage occurs between E17.4 and PND5 [15]. Furthermore, in our hands intra-amniotic LPS combined with hyperoxia commencing from PND3.5 was sufficient to induce BPD-like lung injury as early as PND7 (refer to Additional file 1: Figure S1; see also Figs. 2a and 5a, b).

In this experiment, pups were exposed to either normoxia or hyperoxia on PND0, and intravenous administration of 100,000 hAECs was given either on PND0.5 or on PND4. Pups were culled either on PND7 or PND14 (*n* = 6–8 per group). A representation of the experimental design is shown in Fig. 1c.

The five experimental groups were: (a) intra-amniotic saline + normoxia + intravenous saline; (b) intra-amniotic saline + normoxia + intravenous hAECs; (c) intra-amniotic LPS + hyperoxia + intravenous saline; (d) intra-amniotic LPS + hyperoxia + intravenous early hAECs (PND0.5); and (e) intra-amniotic LPS + hyperoxia + intravenous late hAECs (PND4).

#### Long-term study

Pups were given hAECs intravenously either on PND0.5 or PND4 and were kept in the hyperoxia chamber from PND0 to PND28, when they were weaned and transferred to standard housing facilities. Animals were culled at either 6 or 10 weeks of age (*n* = 5–7 per group). A representation of the experimental design is shown in Fig. 1d.

All of the injections (LPS and hAEC injections) were performed using a programmable microinjector (IM 300; Narishige, Tokyo, Japan) with bevelled borosilicate glass micropipettes (inner diameter 80–100 μm) and visualised under microscopy. Maternal and neonatal well-being was monitored daily. Animals were humanely killed with sodium pentobarbitone (Virbac, Regents Park, NSW, Australia).

### Lung histology

Mouse pup lungs were perfused with saline, and then inflated and fixed with 4% (w/v) paraformaldehyde at a pressure of 12 cmH_2_O. Paraffin sections were cut at 5-μm thickness and stained with haematoxylin and eosin (H&E). Eight to 10 non-overlapping sequential fields of view were taken at 200× magnification. Lung development and alveolar simplification were determined by measuring the tissue-to-airspace ratio as described previously [8]. Briefly, ImageJ software was used to set thresholds that differentiated between the lung tissues and alveolar space. The areas of lung tissues and airspace were then obtained for the calculation of the ratio of tissue to airspace.

### Multikine ELISA for inflammatory cytokines

Protein was isolated from snap-frozen lung tissue. Assessment of inflammatory cytokine concentration was performed using a Multiplex ELISA specific for mouse cytokines (Bio-rad, CA, USA). The levels of cytokines were determined by the Luminex 200 System (Luminex Corp., Austin, TX, USA), and normalised against total protein concentration.

### Flow cytometry analysis of lung immune cell populations

**Fig. 3:**
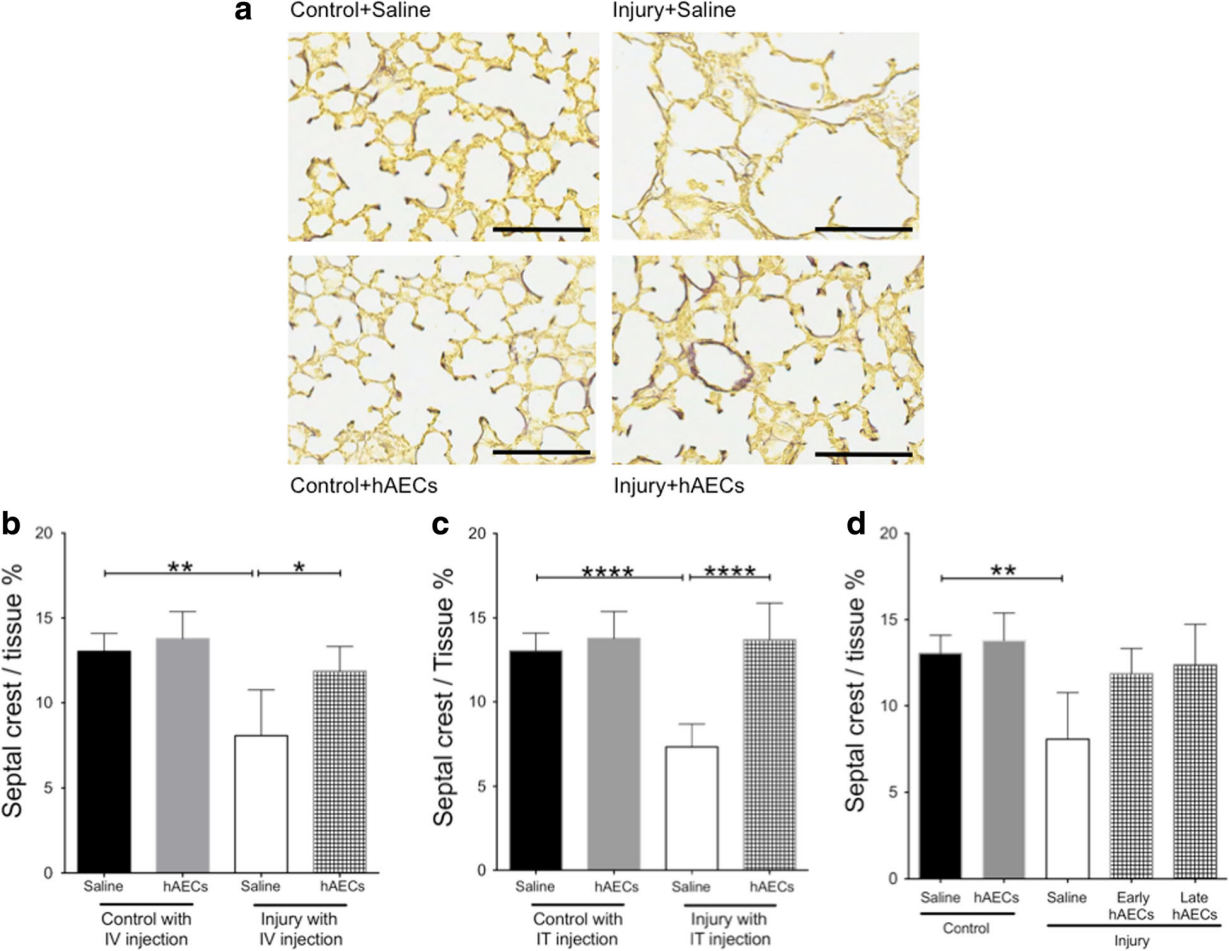
Hart’s staining on PND14 (*n* = 6–8). **a** Representative images for Hart’s staining. Scale bar = 100 μm. **b**, **c** Secondary septal crest density was decreased in the saline-treated injured group. Early intravenous (**b**) and intratracheal (**c**) injection of hAECs restored the tissue-to-air space ratio. **d** Both early and late treatment restored septal crest density. Data expressed as mean ± standard error of mean (SEM). Statistical significance determined with one-way ANOVA accompanied by the Bonferroni post-hoc test. *p < 0.05, **p < 0.01, ****p < 0.0001. hAEC human amnion epithelial cell, IT intratracheal, IV intravenous

For flow cytometry experiments, pups were culled on day 7 (*n* = 6) and whole lungs were minced mechanically before digestion with collagenase D (1 mg/ml) at 37 °C for 20 minutes. Lung homogenate was passed through 70-μm and 40-μm filters to obtain a single cell suspension prior to staining. After blocking Fc receptors, cells were stained using a combination of surface markers and intracellular stains. These included CD45, CD25, NKp46, Foxp3, MHCII and F4/80 purchased from eBioscience, CD4, CD3, CD8, B220 and CD11c from Biolegend, and CD11b, CD103, Ly6C, Ly6G and SiglecF from BD. A full list of antibodies is presented in Table 1. Natural killer cells were identified as the CD45^+^NKp46^+^CD3^–^ population, neutrophils were identified as the CD45^+^Ly6G^hi^CD11b^hi^CD11C^hi^F4/80^–^ population, CD11bc dendritic cells were identified as the CD45^+^CD103^lo^CD11C^hi^Ly6C^–^F4/80^int^MHCII^hi^CD11b^hi^Siglec-F^–^ population, CD103c dendritic cells were identified as the CD45^+^CD103^hi^CD11C^hi^Ly6C^–^F4/80^lo^MHCII^hi^CD11b^lo^Siglec-F^–^ population and interstitial macrophages (imacs) were identified as the CD45^+^CD103^lo^CD11C^lo^Ly6C^lo^F4/80^int^MHCII^hi^CD11b^lo^Siglec-F^–^ population. The representative FACS plots for these populations are shown in Fig. 3a, d, f. Analysis was performed using a BD FACS Canto II Flow (BD Biosciences, NSW, Australia).

**Table 1:** Antibody information for fluorescence-activated cell sorting analysis

Antibody	Manufacturer	Catalogue number
Ly6C-BV605	BD Biosciences	563011
SiglecF-PE	BD Biosciences	562068
Ly6G-AF700	BD Biosciences	561236
CD103-BV510	BD Biosciences	563087
F4/80-FITC	Jomar Life Research	11-4801-85
CD45-PE Cy7	eBioscience	25-0451-81
CD4-PE	eBioscience	12-0041-83
CD11b-PerCP Cy5.5	eBioscience	45-0112-82
CD3-BV421	Biolegend	100227
CD8-BV510	Biolegend	100752
CD25-APC Cy7	Biolegend	102206
NKp46-BV605	Biolegend	137619
B220-APC	Biolegend	103211
Foxp3-FITC	Biolegend	320012
CD11c-BV421	Biolegend	107645
I-A/I-E-BV785	Biolegend	117330

### 3D bronchoalveolar and type II alveolar cell cultures

BASCs and type II alveolar (AT2) cells were isolated from pups on PND14 (*n* = 4) as described previously [16]. Briefly, lung tissues were digested and a single cell suspension was obtained before staining and cell sorting using a BD Influx system (BD Biosciences). The cells were stained using the following antibody panel: CD45-AF647, CD31-FITC, EpCAM-PE, Sca1 (Ly-6A/E)-BV421 from BD Biosciences with propidium iodide (PI) for exclusion of dead cells. BASCs were gated as the CD45^neg^CD31^neg^Sca-1^pos^EpCAM^pos^ population, and AT2 cells were gated as the CD45^neg^CD31^neg^Sca-1^neg^EpCAM^pos^ population (Fig. 8a).

Freshly isolated AT2 cells and BASC cells (2000 cells per well) from each experimental group were mixed with Matrigel containing mouse endothelial cells (20,000 cells per well). Cells were co-cultured and plated in triplicate using transwell inserts (6.5 mm, pore size 5.0 μm). Images were taken after 2 weeks in culture, and colonies picked after 3 weeks in culture for immunohistochemical analysis. Cultured AT2 cell and BASC colonies were fixed with 4% (w/v) paraformaldehyde for 30 minutes at room temperature and then immobilised with HistoGel (Thermo Scientific, MA, USA) for paraffin embedding. Colony sections were then deparaffinised in xylene and rehydrated before staining for BASC and AT2 cells.

### Immunohistochemical staining

Paraffin-embedded lung sections were cut at a thickness of 5 μm and deparaffinised in xylene and rehydrated. Hart’s and von Willebrand factor (vWF) staining were performed to stain for elastin and image analysis was completed as described previously [8, 9]. Eight to 10 random images were taken at 200× magnification. The ratio of secondary septal crests to tissue was calculated using ImageJ (National Institute of Health, Bethesda, MD, USA). Pulmonary vessel numbers were determined by counting the number of vWF-positive vessels stratified by diameter (< 50 and 50–100 μm) using ImageJ.

Pulmonary artery thickness was assessed by alpha-smooth muscle actin (α-SMA) staining. Slides were subjected to citrate buffer (pH 6.0) antigen retrieval, followed by incubation with rabbit polyclonal anti-α-SMA antibody (1:100, ab5694; Abcam, UK) overnight at 4 °C. Secondary antibody (anti-rabbit 568, 1:200, A10042; Life Technologies, USA) was applied to the sections for 1 hour at room temperature, followed by nuclear staining with DAPI (Sigma). Fifteen to 20 random images were taken at 200× magnification. Each artery was identified individually by a blinded assessor. The average fluorescence intensity of α-SMA-stained blood vessels was determined by calculating the fluorescence intensity divided by the perimeter of the vessel (ImageJ).

BASCs are located in the terminal bronchioles and AT2 cells are located throughout the parenchyma. BASCs are positive for both pro-surfactant protein C (pro-SPC) and Clara Cell 10 kD protein (CC10) staining while AT2 cells are pro-SPC positive but CC10 negative. Staining was performed as described previously [17]. Briefly, slides were subjected to PBS with 0.2% Triton-X antigen retrieval. After blocking with 10% donkey serum, slides were incubated with goat anti-mouse CC10 antibody (1:100, sc9772; Santa Cruz Biotechnology Inc., Soquel, CA, USA) and rabbit anti-mouse pro-SPC antibody (1:100, ab3786; Abcam, Cambridge, MA, USA) at 4 °C overnight. Secondary antibodies anti-rabbit 568 (1:200, A10042; Life Technologies, Gaithersburg, MD, USA) and anti-goat 488 (1:200, A21467; Life Technologies) were applied to the sections for 1 hour at room temperature followed by nuclear staining with DAPI (Sigma). The average numbers of BASCs per terminal bronchiole were determined by manual counting while the percentage of type II alveolar cells was determined using Imaris software (Bitplane, Zurich, Switzerland).

### Lung function measurement

Invasive plethysmography was performed using the flexiVent system (SCIREQ, Montreal, QC, Canada). Mice were anaesthetised with ketamine/xylazine (200 μl of 100 mg/ml ketamine and 20 mg/ml xylazine per 15 g body weight) and tracheotomised using an 18G cannula, which was connected to the Y-tubing of the plethysmograph. The immediate (baseline) ventilation for each mouse consisted of a tidal volume of 10 ml/kg at 120 breaths/minute respiratory rate with 3 cmH_2_O positive end-expiratory pressure and allowed to stabilise for 5 minutes. Saline or methacholine (3.125, 6.25, 12.5, 50 and 100 mg/ml; Sigma) was then delivered into the trachea from the ventilator through an ultrasonic nebuliser (Aeroneb Lab, standard mist model; Aerogen Ltd, Ireland). We then measured changes to respiratory system resistance (Rrs), compliance (Crs) and pressure–volume loop (PV loop). Resistance measurements were made using a 1.25-s, 2.5-Hz volume-driven oscillation applied to the airways by a computer-controlled piston (SnapShot perturbation). The maximum *R* value with a coefficient of determination of 0.9 or greater was used to determine the dose–response curve. The PV loop was generated from the area under the inflation limb of a 30 ml/kg (three times tidal volume) dynamic PV loop and normalised by the maximum loop volume.

### Echocardiography

Mice were anaesthetised with isoflurane at 3% and maintained at 1.5–2% to reduce the heart rate to within the 400–450 bpm range. Transthoracic echocardiography was performed using a Vevo 2100 (Visualsonics, Toronto, Canada) and a 40-MHz linear transducer with simultaneous ECG recording. In the anteriorly angulated left parasternal long-axis view, PW Doppler was applied to measure the pulmonary artery acceleration time (PAT) and the pulmonary artery ejection time (PET). M-mode was applied to determine right ventricle anterior wall thickness (RVAWT).

### Statistics

Investigators were blind to the experimental groups during the analysis. Data are expressed as mean ± standard error of mean (SEM). Statistical significance was determined using GraphPad Prism (GraphPad Software Inc., San Diego, CA, USA) with one-way ANOVA accompanied by the Bonferroni post-hoc test for multiple groups. Statistical significance was accorded when *p* < 0.05.

## Results

### Neonatal outcomes

Antenatal inflammation and postnatal hyperoxia have been associated previously with reduced body weights [18]. This was consistent with our observations at PND14 in this study. Notably, the weight loss associated with the experimental injury was not observed in either hAEC treatment group (Table 2). Differences in organ weights (normalised against body weights) were also not observed between groups at either time point (Tables 3 and 4).

**Table 2:** Pups’ body weight on PND7 and PND14

Group	Body weight (g)	
	PND7	PND14
Control + saline	3.41 ± 0.13	7.16 ± 0.38
Control + hAECs	3.10 ± 0.12	6.06 ± 0.22
Injury + early treatment		
Saline	3.27 ± 0.16	4.86 ± 0.21****
hAECs	2.90 ± 0.10	6.71 ± 0.10
Injury + late treatment		
Saline	2.95 ± 0.10	4.65 ± 0.31****
hAECs	3.15 ± 0.18	5.69 ± 0.15**

*hAEC* human amnion epithelial cell, *PND* postnatal day

**p < 0.01, ****p < 0.0001

**Table 3:** Pups’ organ weight/body weight on PND7

Group	Organ weight/body weight (%)				
	Lung	Heart	Liver	Kidney	Brain
Control + saline	2.96 ± 0.10	0.77 ± 0.06	3.49 ± 0.23	1.28 ± 0.07	6.51 ± 0.25
Control + hAECs	3.39 ± 0.09	0.64 ± 0.02	3.14 ± 0.09	1.32 ± 0.07	7.80 ± 0.18
Injury + early treatment					
Saline	2.85 ± 0.21	0.60 ± 0.04	3.43 ± 0.07	1.28 ± 0.03	6.92 ± 0.25
hAECs	3.21 ± 0.19	0.72 ± 0.06	3.25 ± 0.13	1.27 ± 0.06	7.43 ± 0.39
Injury + late treatment					
Saline	3.03 ± 0.13	0.63 ± 0.07	3.86 ± 0.19	1.31 ± 0.05	6.48 ± 0.16
hAECs	3.13 ± 0.18	0.75 ± 0.09	3.45 ± 0.20	1.39 ± 0.04	7.24 ± 0.22

*hAEC* human amnion epithelial cell, *PND* postnatal day

**Table 4:** Pups’ organ weight/body weight on PND14

Group	Organ weight/body weight (%)				
	Lung	Heart	Liver	Kidney	Brain
Control + saline	2.51 ± 0.09	0.68 ± 0.02	3.80 ± 0.31	1.35 ± 0.06	5.16 ± 0.25
Control + hAECs	2.70 ± 0.06	0.80 ± 0.03	2.87 ± 0.08	1.23 ± 0.05	6.38 ± 0.14
Injury + early treatment					
Saline	2.79 ± 0.15	0.67 ± 0.03	3.41 ± 0.21	1.42 ± 0.04	5.84 ± 0.54
hAECs	2.49 ± 0.10	0.66 ± 0.02	3.65 ± 0.23	1.05 ± 0.10	5.51 ± 0.12
Injury + late treatment					
Saline	2.95 ± 0.17	0.68 ± 0.05	3.26 ± 0.16	1.42 ± 0.04	7.96 ± 0.50
hAECs	2.82 ± 0.14	0.78 ± 0.04	3.42 ± 0.31	1.53 ± 0.08	6.46 ± 0.30

*hAEC* human amnion epithelial cell, *PND* postnatal day

#### hAEC administration improved lung tissue-to-air space ratio and secondary septal crest density

To examine the efficacy of hAECs in experimental BPD we first assessed lung pathology. Alveolar simplification is a characteristic pathology of BPD where the lung parenchyma has fewer and larger alveoli [3], reducing the tissue-to-air space ratio. In this study, injured animals (intra-amniotic LPS + hyperoxia) had significantly reduced tissue-to-air space ratio (*p* < 0.0001) (Fig. 2a–e and Additional file 1: Figure S1) compared to healthy controls. This was mitigated by the two highest doses of hAECs (75,000 and 100,000 hAECs) by PND7 (*p* < 0.01 and *p* < 0.001 respectively) in a dose-dependent manner (Fig. 2a). Both intravenous and intratracheal administration of hAECs restored the tissue-to-air space ratio by PND14 with no significant differences between the routes of administration (Fig. 2b, c). With regards to the timing of hAEC administration, we found that the tissue-to-air space ratio was improved in both early and late hAEC treatment groups (*p* < 0.01, Fig. 2d, e). However, the tissue-to-air space ratio remained significantly lower in the late treatment group compared to healthy controls at PND7 (*p* < 0.01, Fig. 2d) and PND14 (*p* < 0.05, Fig. 2e).

During normal lung development elastin fibres become concentrated at the tips of secondary septal crests [19]. This localisation of elastin is essential for normal lung development, with a failure in elastin deposition resulting in abnormal alveolarisation [20]. Consequently, the number of secondary septal crests with elastin-positive tips is a useful measure of secondary septal crest development. Here we showed that the combination of antenatal inflammation and postnatal hyperoxia significantly decreased secondary septal crest density by PND14 (*p* < 0.01 and *p* < 0.001, Fig. 3a–c) compared to the controls. Both early intravenous and intratracheal administration of hAECs restored the secondary septal crest density with no significant differences between the routes of administration (Fig. 3b, c). Both early and late hAEC administration improved secondary septal crest density with no differences between the times of administration (Fig. 3d). These data showed that hAEC treatment improved the experimental BPD lung architecture by improving/restoring the tissue-to-air space ratio and secondary septal crest density.

#### hAEC treatment decreased lung inflammation

**Fig. 4:**
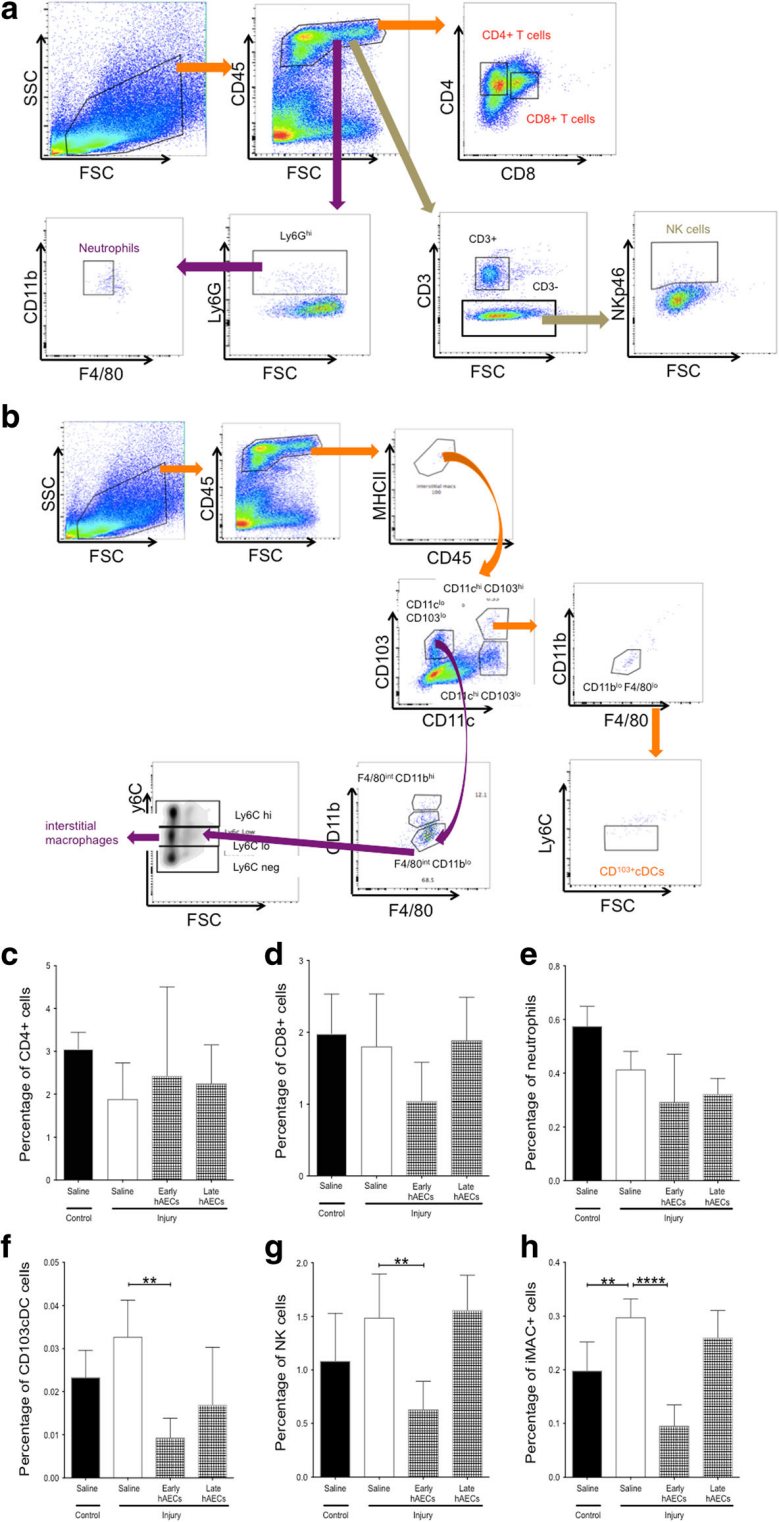
FACS in mouse lung cells on PND7 (*n* = 6–8). **a** FACS gating strategies for CD4^+^ T cells, CD8^+^ T cells, neutrophils (CD45^+^Ly6G^hi^CD11b^hi^CD11C^hi^F4/80^–^) and natural killer cells (CD45^+^NKp46^+^CD3^–^). **b** FACS gating strategies for CD103^+^CD11c^+^ dendritic cells (CD45^+^CD103^hi^CD11C^hi^Ly6C^-^F4/80^lo^MHCII^hi^CD11b^lo^Siglec-F^–^ cells) and interstitial macrophages (CD45^+^CD103^lo^CD11C^lo^Ly6C^lo^F4/80^int^MHCII^hi^CD11b^lo^Siglec-F^–^). **c, d** There were no differences in CD4^+^ and CD8^+^ populations across groups. **e** There was no difference in the percentage of neutrophils between groups. **f**–**h** Percentage of CD103^+^CD11c^+^ dendritic cells (**f**) and natural killer cells (**g**) increased in the injured group, but was only significantly returned to control levels by early treatment. **h** Percentage of interstitial macrophages significantly increased in the injured animals, but was brought back to control levels by early treatment. Data expressed as mean ± standard error of mean (SEM). Statistical significance determined with one-way ANOVA accompanied by the Bonferroni post-hoc test. **p < 0.01, ****p < 0.0001. FSC forward scatter, SSC side scatter, NK natural killer, DC dendritic cell, hAEC human amnion epithelial cell

Next we investigated the anti-inflammatory effect of hAECs in this murine BPD model since pulmonary inflammation is another characteristic of BPD and hAECs have been shown to exert anti-inflammatory effects in other small and large animal models of lung injury [21, 22]. We assessed changes to the immune cell subpopulations and cytokine levels associated with BPD-like injury. Given that hAECs have been reported previously to exert profound immunomodulatory effects in an adult lung injury model, we were surprised to note that there was no change to the proportions of CD4^+^ and CD8^+^ T cells and neutrophils across all experimental groups (Fig. 4a, c–e). However, the percentage of interstitial macrophages was elevated in the injured group (*p* < 0.01, Fig. 4 h), an effect only mitigated by early hAEC administration. The percentage of CD103^+^CD11c^+^ dendritic cells (DCs) and NK cell populations were also increased in the injured groups, a change normalised by early hAEC administration (*p* < 0.01, Fig. 4f, g). Given the critical roles of DCs and NK cells in recognition and removal of dead and dying cells, this observation suggests that only early hAEC administration was able to significantly alter disease progression sufficiently to prevent the activation and recruitment of DCs and NK cells to the lungs.

**Fig. 5:**
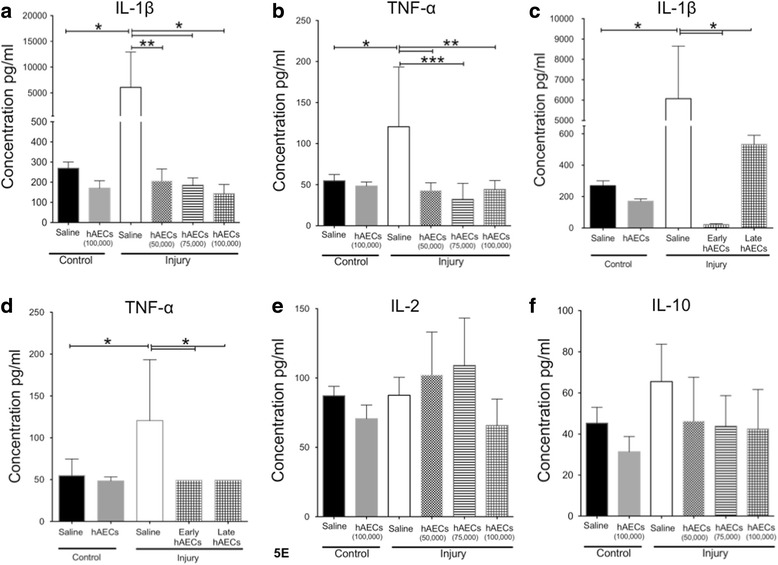
Changes in inflammatory cytokine levels measured from mouse lung on PND7 (*n* = 6–8). In our experience, the detection limits for the cytokines are: IL-1β, 10.36 pg/ml; TNF-α, 5.8 pg/ml; IL-2, 3.72 pg/ml; IL-4, 6.98 pg/ml; IL-6, 0.74 pg/ml; IL-10, 2.95 pg/ml; IFN-γ, 1.84 pg/ml; GM-CSF, 6.2 pg/ml; LIF, 1.75 pg/ml; MIP-2, 6.05 pg/ml; MCP-1, 22.4 pg/ml. IL-1β (**a**) and TNF-α (**b**) levels in the dosage study: both were increased in the injured group, but all dosages of hAECs significantly reduced IL-1β and TNF-α back to control levels. IL-1β (**c**) and TNF-α (**d**) levels in the time-of-dose effect study: both early and late hAEC administration significantly reduced IL-1β and TNF-α back to control levels. IL-2 (**e**) and IL-10 (**f**) levels in dosage groups: there were no differences in IL-2 and IL-10 levels between groups. Data expressed as mean ± standard error of mean (SEM). Statistical significance determined with one-way ANOVA accompanied by the Bonferroni post-hoc test. *p < 0.05, **p < 0.01, ***p < 0.001. hAEC human amnion epithelial cell, IL interleukin, TNF tumour necrosis factor

At PND7 we found that IL-1β and TNF-α were significantly elevated in the injured group (*p* < 0.05). Interestingly, all three dosages of early hAEC administration reduced IL-1β and TNF-α to levels comparable to the control groups (*p* < 0.05 and *p* < 0.01 respectively, Fig. 5a, b). Both early and late hAEC treatment significantly reduced the levels of IL-1β and TNF-α levels comparable to controls (Fig. 5c, d). We did not observe any differences in the levels of IL-2 and IL-10 between groups (Fig. 5e, f). Notably, levels of IL-4, IL-5, IFN-γ and Granulocyte-macrophage colony-stimulating factor (GM-CSF) were below the limit of detection.

**Fig. 6:**
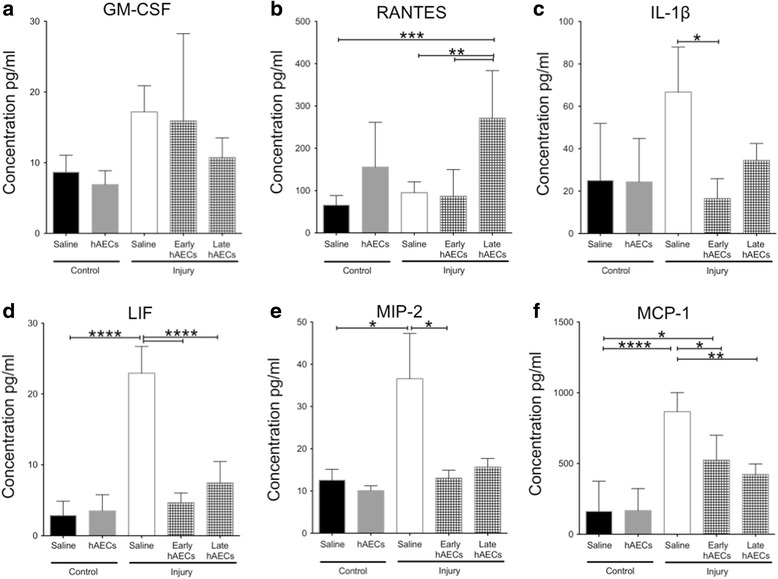
Changes of inflammatory cytokine levels in mouse lung lysate on PND14 (*n* = 6–8). **a** There was no difference in GM-CSF levels across all the groups. **b** Level of RANTES was significantly increased in the late but not the early hAEC treatment group compared to control and injury groups. **c** IL-1β level was increased in the injured group, and was significantly reduced to control levels by early hAEC treatment. **d**–**f** Levels of LIF (**d**), MIP-2 (**e**) and MCP-1 (**f**) were significantly increased in the injured group, but were reduced in both early and late treatment groups. Data expressed as mean ± standard error of mean (SEM). Statistical significance determined with one-way ANOVA accompanied by the Bonferroni post-hoc test. *p < 0.05, **p < 0.01, ***p < 0.001, ****p < 0.0001. hAEC human amnion epithelial cell, IL interleukin, LIF leukemia inhibitory factor, MIP macrophage inflammatory protein, MCP monocyte chemoattractant protein

By PND14 there was no significant difference in GM-CSF levels between groups (Fig. 6a). The level of Regulated on Activation Normal T cell Expressed and Secreted (RANTES) was elevated in the late hAEC treatment group but not the early treatment group (Fig. 6b, *p* < 0.001). Injury increased levels of LIF (*p* < 0.0001, Fig. 6d), MIP-2 (*p* < 0.05, Fig. 6e) and MCP-1 (*p* < 0.001, Fig. 6f) by PND14, and this was mitigated in both early and late treatment groups. The level of IL-1β was increased in the injured group, but this was not statistically significant. Regardless, early hAEC treatment significantly reduced IL-1β levels (*p* < 0.05, Fig. 6c). Levels of TNF-α and IL-6 were below the limit of detection. These results suggest that hAECs were anti-inflammatory in this BPD model. Although both early and late hAEC treatments were able to decrease the inflammatory cytokines, the changes in immune cell subpopulations were only observed in the animals that received hAECs early.

#### hAEC treatment increased the number of vWF-positive pulmonary vessels

**Fig. 7:**
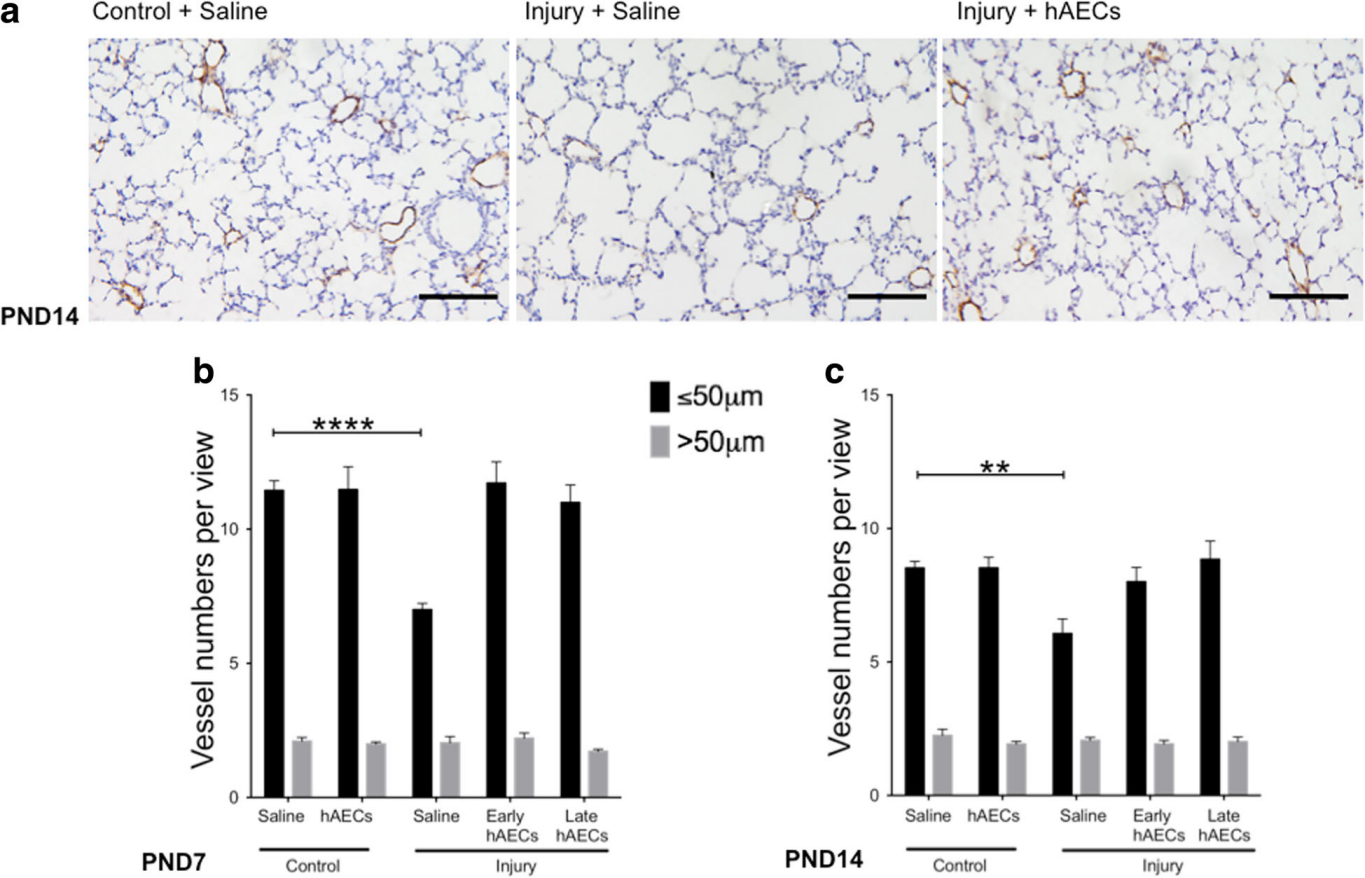
vWF immunohistochemistry in mouse lung tissue at PND7 and PND14 (*n* = 6–8). **a** Representative images for vWF staining on PND14. Scale bar = 100 μm. **b**, **c** Average number of vessels per field of view on PND7 (**b**) and PND14 (**c**). Average number of vessels with diameter < 50 μm was decreased in saline-treated injured mice, which was reversed after hAEC treatment on both PND7 and PND14. Black bars refer to vessels < 50 μm and grey bars refer to vessels > 50 μm. There was no difference in the numbers of larger blood vessels (i.e. > 50 μm) between groups. Data expressed as mean ± standard error of mean (SEM). Statistical significance determined with one-way ANOVA accompanied by the Bonferroni post-hoc test. **p < 0.01, ****p < 0.0001. hAEC human amnion epithelial cell, PND postnatal day

Angiogenesis is essential for normal lung development and is closely tied with alveolar development and maintenance of alveolar structures in the developing lung [23]. As such, we sought to assess whether angiogenesis was disrupted in our experimental model of BPD and whether hAECs were able to rescue that. Here we report that our model of BPD-like lung injury significantly decreased the numbers of smaller pulmonary blood vessels (< 50 μm in diameter) on both PDN7 and PDN14 (*p* < 0.0001 and *p* < 0.01 respectively, Fig. 7a–c). Both early and late hAEC treatment restored blood vessel numbers to control levels (Fig. 7b, c). Larger blood vessels (> 50 μm) were unaffected across all experimental groups. This suggests that hAEC treatment may improve alveolar development partly by promoting pulmonary angiogenesis and increasing the density of the pulmonary capillary bed.

#### hAECs induced bronchioalveolar stem cell but not type II alveolar cell proliferation in vivo

**Fig. 8:**
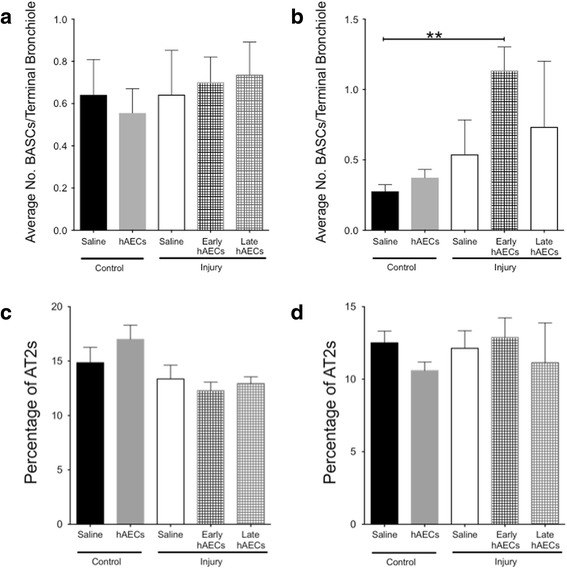
BASC and AT2 cells in neonatal mouse lungs at PND7 and PND14 (*n* = 6–8). **a, b** Average number of BASCs per terminal bronchiole on PND7 (**a**) and PND14 (**b**). There was no difference in the average number of BASCs per terminal bronchiole on PND7. This number was increased by early hAEC treatment, although there was no difference in late treatment group and injured groups on PND14. **c**, **d** Percentage of AT2 cells on PND7 (**c**) and PND14 (**d**). There was no difference in the percentage of AT2 cells between groups at both time points. Data expressed as mean ± standard error of mean (SEM). Statistical significance determined with one-way ANOVA accompanied by the Bonferroni post-hoc test. **p < 0.01. AT2s type II alveolar cells, BASC bronchioalveolar stem cell, hAEC human amnion epithelial cell

Low engraftment rates of hAECs led us to postulate that their beneficial effects may be associated with an activation of endogenous stem/progenitor cells in the lungs [8, 24]. We were specifically interested in bronchioalveolar stem cells (BASCs) and type II alveolar (AT2) cells given previous work implicating their involvement in alveolar epithelial repair [25]. AT2 cells are lung progenitor cells that possess the capacity to proliferate and differentiate into AT1 cells following injury [26, 27]. BASCs are more recently described lung stem/progenitor cells that have been demonstrated to self-renew and differentiate into both AT2 and AT1 cells in the distal lung [28]. BASCs were identified at the terminal bronchioles by immunohistochemical staining for pro-SPC and CC10 (Additional file 1: Figure S2). The average number of BASCs per terminal bronchiole is indicative of the activation status of the niche [16]. Here we found that the BASC niches remained quiescent even in the injured animals (Fig. 8a, b). However, the average numbers of BASCs were elevated in both early and late hAEC treatment groups. This effect was greater in the animals that received hAECs earlier (*p* < 0.05, Fig. 8b). No differences in the percentage of AT2 cells were observed between experimental groups at either PND7 or PND14 (Fig. 8c, d). These observations suggest that activation of the BASC niche may account for some of the pro-regenerative effects of hAECs in a BPD-like lung injury setting.

**Fig. 9:**
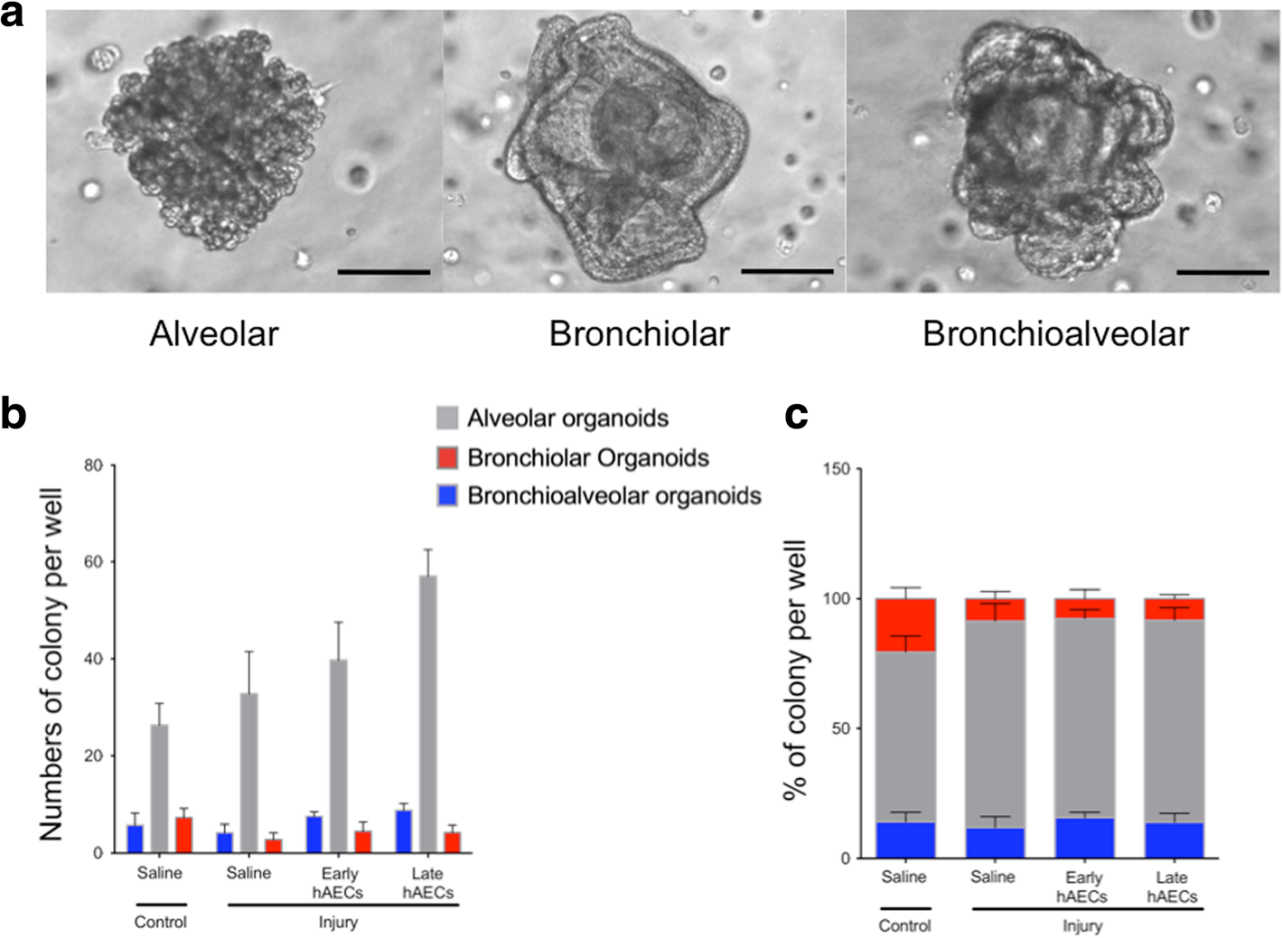
BASCs and AT2 cells in in-vitro culture (*n* = 4–5). **a** Representative images of alveolar, bronchiolar and bronchioalveolar organoids after 2 weeks of culture. Scale bar = 100 μm. **b**, **c** Size (**b**) and percentage (**c**) of each type of colony. There were no differences in the size and the percentage of each type of colony across all experimental groups. Data expressed as mean ± standard error of mean (SEM). Statistical significance determined with one-way ANOVA accompanied by the Bonferroni post-hoc test. hAEC human amnion epithelial cell

Alveologenesis refers to the normal development of alveoli and is a critical part of maintaining existing lung architecture [23]. This process is dovetailed with angiogenesis in the lung where endothelial cells serve as the stromal cells that support alveologenesis. This relationship between stem/progenitor cells and stromal cells is recapitulated in the 3D organoid cultures where the endothelial cells were shown to significantly influence the growth characteristics of BASCs and AT2 cells [16]. In light of this, we asked whether the activation of the stem cell niche was due to a positive impact of hAECs on BASCs or their supporting endothelial cells. To this end, we isolated primary cells from mouse lungs representative of each experimental group and cultured them as organoids supported by lung endothelial cells from healthy mice, thereby controlling for the contribution of the stroma (Additional file 1: Figure S3A). Organoids with defined structures were observed after 2 weeks in culture. In line with previous reports [16], AT2 cells were only capable of forming alveolar structures while BASCs could give rise to organoids with three distinct phenotypes: alveolar, bronchiolar and bronchioalveolar (Fig. 9a). In keeping with their morphology, alveolar organoids expressed pro-SPC, bronchiolar organoids expressed CC10 and bronchioalveolar organoids were found to express pro-SPC and CC10 (Additional file 1: Figure S3B). No differences in the size, number and percentage of each type of colony were seen across all the experimental groups (Fig. 9b, c).

#### hAEC treatment reduced peripheral pulmonary arterial remodelling

**Fig. 10:**
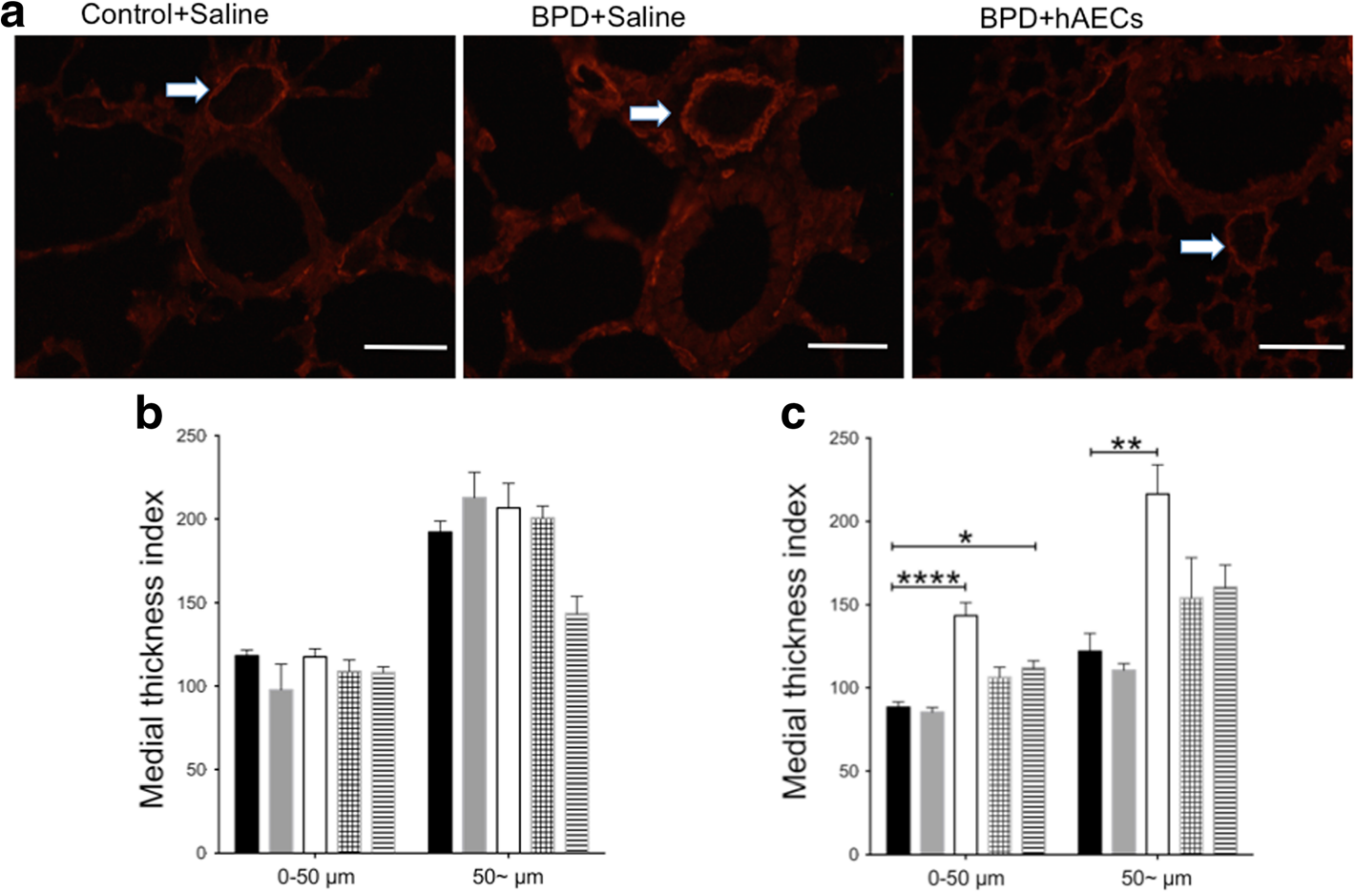
α-SMA immunofluorescence in mouse lung tissue at PND7 and PND14 (*n* = 6–8). **a** Representative images for α-SMA immunofluorescence in mouse lung tissues at PND14. Scale bar = 50 μm. Vessels indicated with white arrows. **b** There were no detectable differences in the arterial medial thickness at PND7. **c** By PND14, arterial medial thickness was increased in injured mice. This was mitigated by both early and late hAEC treatment. Data expressed as mean ± standard error of mean (SEM). Statistical significance determined with one-way ANOVA accompanied by the Bonferroni post-hoc test. *p < 0.05, **p < 0.01, ****p < 0.0001. BPD bronchopulmonary dysplasia, hAEC human amnion epithelial cell

Persistent pulmonary hypertension and right heart failure occur at the end stages of chronic lung diseases including BPD [29]. Before pulmonary hypertension develops, the peripheral pulmonary arteries become stiffer and thicker, as a result of vascular muscularisation. In light of this, we investigated the potential for this secondary complication to arise in this model of experimental BPD. We measured the thickness of the arterial medial layer and found that the medial thickness of pulmonary arteries was unchanged at PND7 (Fig. 10b) but increased in the injury group by PND14. This was attenuated by both early and late hAEC treatment. However, this effect was more pronounced in the early group (*p* < 0.01, Fig. 10a, c).

### Long-term outcomes

It was important for us to assess long-term outcomes in our model of experimental BPD in order to assess its relevance to the clinical condition and infer the likelihood of hAEC utility in this disease setting. To this end, pups were maintained in hyperoxia until they were weaned and transferred to normoxia at 4 weeks of age. Their respiratory and cardiovascular function was then examined either at 6 or 10 weeks of age, equating to periods of adolescence and early adulthood, when clinical respiratory and cardiovascular complications of BPD are recognised [30–32].

#### Early hAEC treatment improved lung tissue-to-air space ratio

**Fig. 11:**
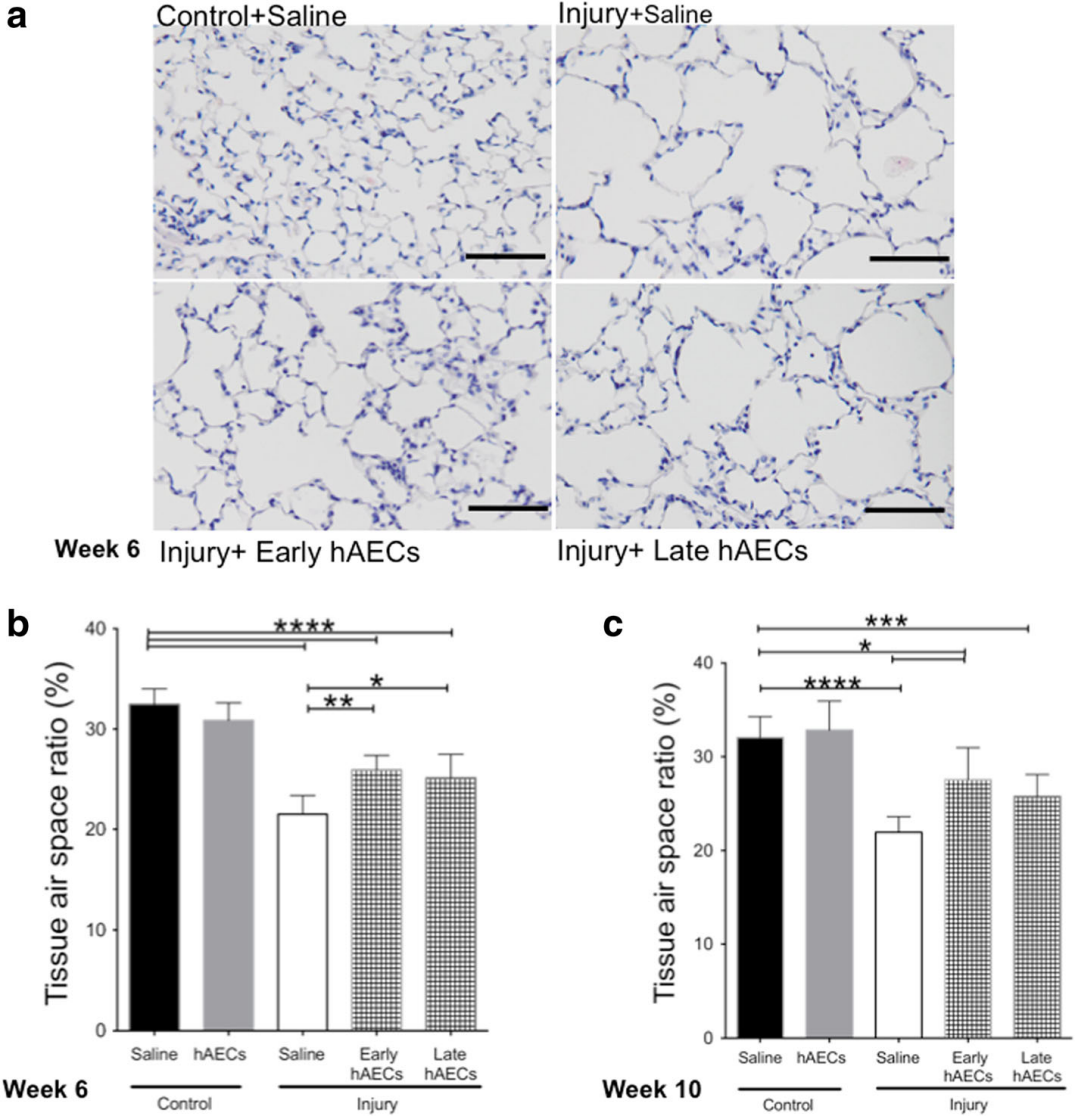
H&E staining of mouse lung tissue at week 6 and 10 (*n* = 5–7). **a** Representative images for H&E staining on mouse lung tissues by week 10. Scale bar = 200 μm. **b** By week 6, both early and late hAEC treatment improved the tissue-to-air space ratio compared to saline-treated injured mice, but remained lower than controls. **c** By week 10, only early hAEC treatment significantly improved the tissue-to-air space ratio, while late treatment made no significant difference to the saline-treated injured mice. Data expressed as mean ± standard error of mean (SEM). Statistical significance determined with one-way ANOVA accompanied by the Bonferroni post-hoc test. *p < 0.05, **p < 0.01, ***p < 0.001, ****p < 0.0001. hAEC human amnion epithelial cell

We observed that injury induced by the combination of antenatal inflammation and postnatal hyperoxia resulted in sustained alveolar simplification seen in the reduced tissue-to-airspace ratio even after 6 weeks of recovery in normoxia at 10 weeks of age (Fig. 11a, c). Improvements achieved in both early and late hAEC treatment groups were sustained at 6 weeks of age (Fig. 11b). Notably, improvement in the tissue-to-airspace ratio was sustained in the early treatment group until 10 weeks of age. This effect was not as long lasting in the late treatment group (Fig. 11c).

#### Early hAEC treatment prevents the increase of airway responsiveness

**Fig. 12:**
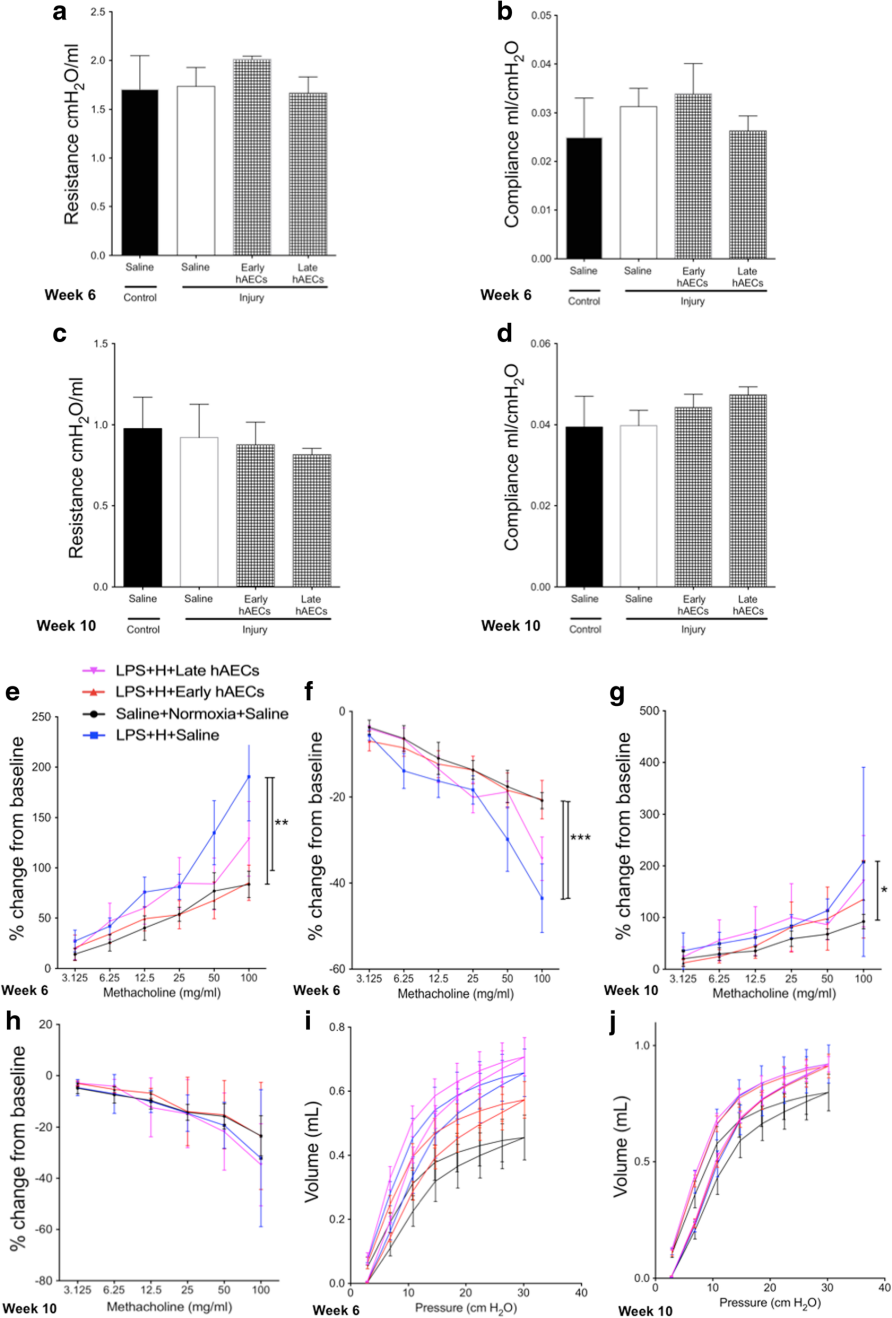
Invasive lung function test in 6-week-old and 10-week-old mice (*n* = 5–7). **a**–**d** There were no significant differences in the baseline of either Rrs at 6 weeks (**a**) and 10 weeks (**c**) or Crs at 6 weeks (**b**) and 10 weeks (**d**) between groups at both time points. **e**, **f** By week 6, compared to control group, Rrs was significantly increased (**e**) and Crs was significantly decreased (**f**) at the 100 mg/ml methacholine dose in the injured group, but this effect was diminished with early hAEC treatment and also improved slightly in the late treatment groups. **g** By week 10, Rrs was significantly increased at 100 mg/ml methacholine dose in the injured group compared to the control group; both treatment groups did not significantly affect Rrs. **h** Crs did not change significantly across all experimental groups. **i**, **j** PV loop at week 6 (**i**) and week 10 (**j**). There was a significant upward shift of the PV loop in the injured group compared to the control group at both time points. By week 6, while the PV loop of the early hAEC treatment group was intermediate to both control and saline-treated injured groups, late treatment had no effect on the position of the PV loop. However, the PV loop position was not altered by either early or late hAEC treatment in week 10. Data expressed as mean ± standard error of mean (SEM). Statistical significance determined with one-way ANOVA accompanied by the Bonferroni post-hoc test. *p < 0.05, **p < 0.01, ***p < 0.001. hAEC human amnion epithelial cell, LPS lipopolysaccharide, H hyperoxia

We next sought to assess lung function in adult animals to determine whether the impact of hAECs to lung structure corresponded with long-term improvements in respiratory function. Using invasive plethysmography we first confirmed using aerosolised saline that the respiratory system resistance (Rrs) and compliance (Crs) baselines did not significantly differ between groups at week 6 and week 10 (Fig. 12a–d). However, upon stimulation with methacholine at its highest dose (100 mg/ml) the injured group showed significantly increased Rrs and decreased Crs compared to controls by week 6 (*p* < 0.01 and *p* < 0.001 respectively, Fig. 12e, f). This was in line with a recent report that small airway hyper-responsiveness is associated with impaired alveolar development [33]. Early hAEC treatment decreased the Rrs and increased Crs to control levels. Late hAEC treatment also mitigated the airway response, but to an extent that was between the injured and control groups. By week 10, injury significantly increased Rrs compared to controls (*p* < 0.05, Fig. 12g); however, the improvements seen at week 6 did not persist to this point, such that there were no significant differences in the changes of Crs across all the groups by week 10 (Fig. 12h).

We then generated pressure–volume loops (PV loops) by dynamically inflating and deflating the lungs to establish a standard respiratory cycle volume. PV loops offer information about the way the lungs deform during breathing in health and disease. A significant upward shift of the PV loop was observed in the injured group compared to the control group at both week 6 and week 10, indicating greater lung compliance in injured animals. The PV loop of animals that received early hAEC treatment was positioned between the control and saline-treated injured groups. No change was observed in the animals that received the late treatment (Fig. 12i). In keeping with the Rrs and Crs findings, the PV loop position was unchanged in either treatment group by week 10 (Fig. 12j).

#### Early hAEC treatment prevents pulmonary hypertension and right ventricular hypertrophy

**Fig. 13:**
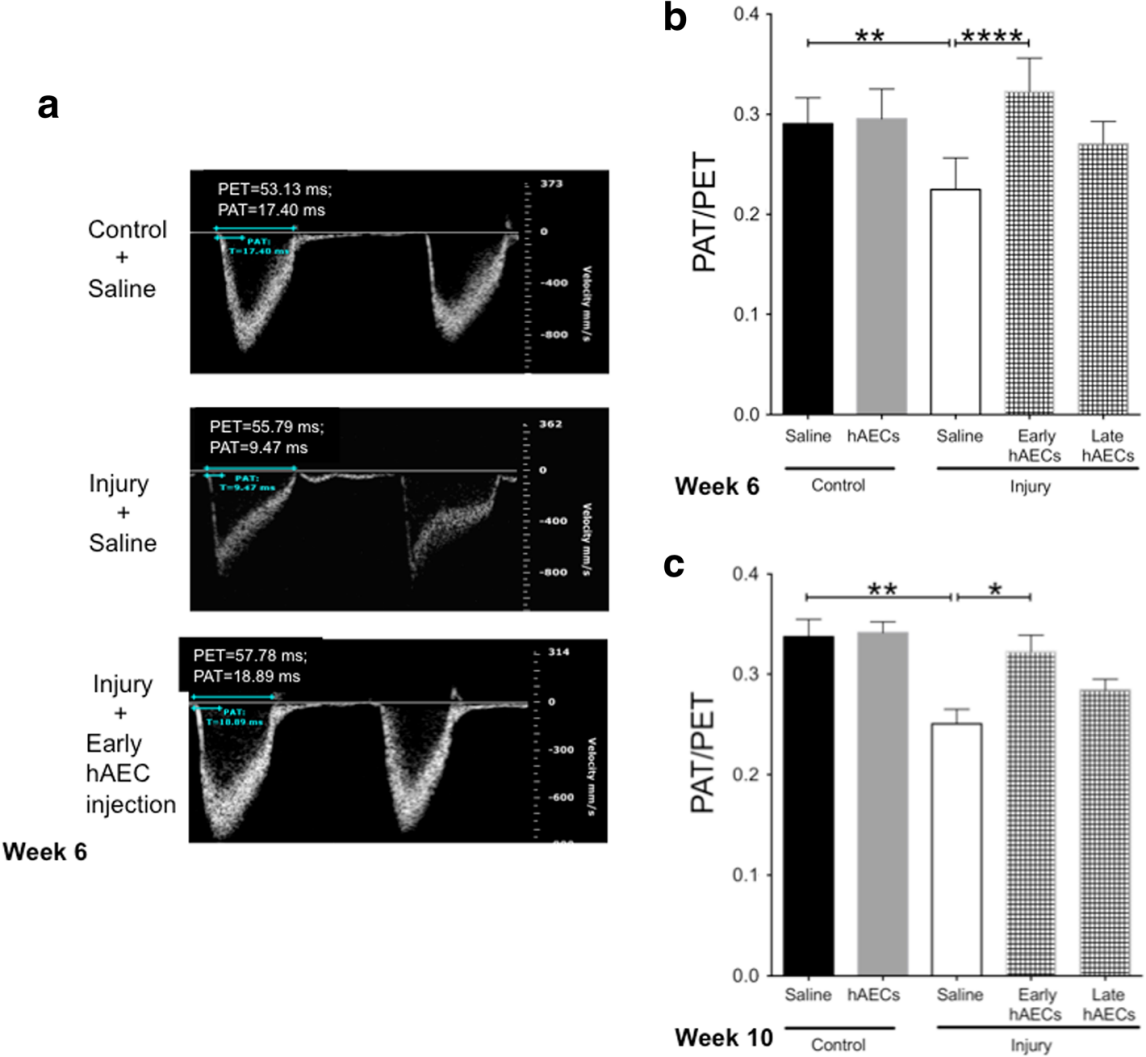
Changes in pulmonary artery flow in 6-week-old and 10-week-old mice (*n* = 5–7). **a** Representative images of pulmonary artery flow by week 6. **b**, **c** Changes of pulmonary artery acceleration time/ejection time (PAT/PET) ratio at week 6 (**b**) and week 10 (**c**). Injury decreased the ratio of PAT/PET on both time points, which indicated the development of pulmonary hypertension. Early hAEC treatment restored the ratio back to control level; however, later treatment attenuated the ratio to be in the middle of the control and injury groups. Data expressed as mean ± standard error of mean (SEM). Statistical significance determined with one-way ANOVA accompanied by the Bonferroni post-hoc test. *p < 0.05, **p < 0.01, ****p < 0.0001. hAEC human amnion epithelial cell

**Fig. 14:**
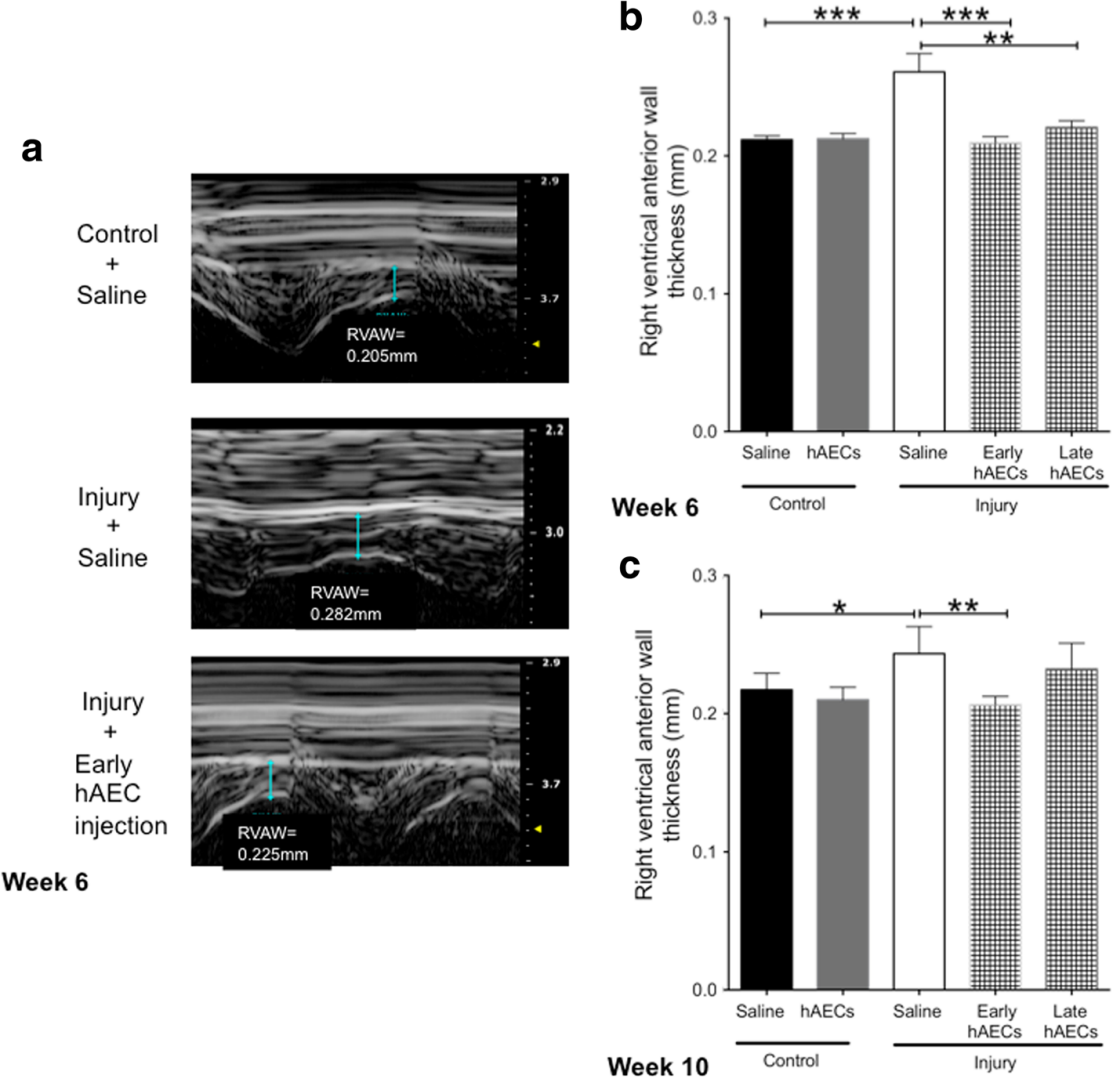
Changes of RVAWT in 6-week-old and 10-week-old mice (*n* = 5–7). **a** Representative images of the RVAWT by week 6. In both week 6 (**b**) and week 10 (**c**) the RVAWT increased in the injured mice; while both hAEC treatment groups decreased the wall thickness to control levels by week 6, only early hAEC treatment mitigated it by week 10. Data expressed as mean ± standard error of mean (SEM). Statistical significance determined with one-way ANOVA accompanied by the Bonferroni post-hoc test. *p < 0.05, **p < 0.01, ***p < 0.001. hAEC human amnion epithelial cell

In order to confirm whether early peripheral pulmonary artery muscularisation leads to pulmonary hypertension later in life we performed echocardiography on mice at 6 and 10 weeks of age. Using echocardiography we confirmed that the injured animals developed pulmonary hypertension by week 6 and this remained evident at week 10, as shown by the reduced pulmonary artery acceleration to ejection time (PAT/PET, Fig. 13a–c). While this effect was attenuated in both treatment groups, we only observed significant improvement in the early treatment group. Furthermore, increase in right ventricle anterior wall thickness (RVAWT) was evident in the untreated injury group at week 6 and this was sustained through to week 10 (Fig. 14a–c). This was attenuated in both early and late treatment groups by week 6 (Fig. 14a, b), but this protective effect only persisted until week 10 in animals given early hAEC treatment (Fig. 14c).

## Discussion

Cell therapy has shown great promise as a treatment for BPD with benefits reported in pre-clinical studies using MSCs [34], hAECs [21] and endothelial progenitor cells [35]. hAECs have been shown previously to improve lung architecture in hyperoxia-induced neonatal lung injury [22]. While this might suggest that hAECs could be useful for BPD, until now there has been a lack of sufficient information necessary to inform clinical translation. Specifically, dose effects, timing of cell delivery and routes of administration have not been explored. The long-term effects of hAEC administration had also not been studied. In this study, we used a clinically relevant model to mimic BPD-like lung injury. We used a combined approach for disease genesis that includes prenatal inflammation and sustained postnatal hyperoxia. Using this approach we were able to demonstrate a dose-dependent improvement in the lung tissue-to-airspace ratio in hAEC-treated animals. We showed that both intratracheal and intravenous administration of hAECs were equally efficacious when administered after lung inflammation was established. Furthermore, we demonstrated that hAEC treatment improved lung architecture and reduced the initial spike in IL-1β and TNFα levels regardless of whether it was administered within 12 hours or 4 days of exposure to hyperoxia. These findings were also associated with a mitigation of pulmonary vessel loss and peripheral pulmonary arterial remodelling that is common associated with BPD. Additionally, early hAEC treatment appeared to be better than later administration.

A previous study reported that three repeated high doses of hAECs (1.5 × 10^6^ per dose) delivered intraperitoneally improved lung structure (tissue-to-airspace ratio and secondary septal crest) in hyperoxia-induced lung injury [22]. However, we were able to normalise lung structure using a single dose of 100,000 hAECs administered intratracheally or intravenously, suggesting that these routes of administration may be more suitable in this context. This observation is important given that most extremely preterm infants presenting with severe respiratory distress are likely to be intubated for respiratory support [36], and certainly to be on intravenous fluid support. Furthermore, this single hAEC dose could resolve lung injury regardless of whether cells were administered at the early stages of pulmonary inflammation or after pulmonary inflammation is well underway. Promisingly, these beneficial effects were sustained into adolescence and early adulthood. Given that the only clinical trial assessing hAECs for experimental BPD employs a single dose of hAEC administered intravenously (1 × 10^6^ cells/kg body weight), the dose effects seen in this study suggests that dose escalation studies in future hAEC clinical trials are certainly warranted. Furthermore, the effect of early hAEC treatment preventing experimental BPD progression in this study suggests that early hAEC delivery to infants at high risk of BPD should also be considered in future clinical trials.

The anti-inflammatory effects of hAECs in this setting of experimental BPD were also assessed. Here we report that antenatal inflammation and postnatal hyperoxia increased pulmonary infiltration of CD103^+^CD11c^+^ dendritic cells, NK cells and interstitial macrophages. This finding supports the observations by Nold et al. [37] where antenatal inflammation was induced by maternal systemic injection of LPS and combined with postnatal hyperoxia. In that study the authors observed increased macrophage and dendritic cell infiltration at PND28, suggesting that temporal changes in immune cell recruitment occur throughout postnatal exposure to hyperoxia. Unfortunately, we were unable to comment on changes to the alveolar macrophages in our current study since the bronchioalveolar lavage fluid was collected prior to lung tissue collection for flow cytometric analysis. Nevertheless, the study described here enabled us to chart the ontogeny of lung inflammation. Accordingly, we were able to assess the impact that different timings of administration have on lung injury and repair, in relation to the ontogeny of inflammation. Levels of IL-1β and TNF-α were elevated in the injured group after hyperoxia exposure as observed in other neonatal lung injury studies [8, 37]. This was diminished by 7 days in both early and late treatment groups. By 14 days, IL-1β levels were reduced dramatically but remained higher in the injured group. Similar to a previous finding by Nold et al. [37] we were unable to detect TNF-α by 14 days. Given that IL-1β and TNF-α are mainly secreted from activated macrophages and monocytes [38], and hAECs decreased the infiltration of interstitial macrophages, it was perhaps unsurprising that early administration of hAECs mitigated this effect.

We further observed that the levels of MCP-1 and MIP-2—the rodent homologue of MCP-1—were significantly increased after 14 days of postnatal hyperoxia exposure. This correlates with clinical studies that reported elevated levels of MCP-1 and IL-8 in the lavage fluid of BPD infants [39, 40]. MCP-1 is known to recruit monocytes to sites of inflammation [41], and activated macrophages and monocytes are known to secrete MIP-2/IL-8, which is chemotactic for polymorphonuclear leukocytes [42]. Additionally, LIF which was also elevated by day 14, is a pro-inflammatory cytokine known to potentiate macrophage aggregation and activation in vitro [43]. In this study, we observed that hAEC administration reduced levels of LIF, MCP-1 and MIP-2 regardless of the timing of hAEC administration. Together, these findings indicate that macrophages are likely a key population through which hAECs mediate neonatal lung repair. Interestingly, RANTES, a cytokine released by T cells and associated with reduced risk of clinical BPD [44], was significantly elevated in the late hAEC treatment group, suggesting that hAECs may be beneficial even after lung injury is underway. It is unclear why RANTES was not elevated in the early hAEC treatment group. One possible explanation is that we may have missed the critical window for measuring this elevation in RANTES levels, given a previous report that RANTES was significantly higher in preterm babies with reduced risk of BPD on PND7, but not PND14 [45]. Future studies using experimental models of BPD should include detailed studies into the relationship between RANTES and severity of lung disease, and should assess its potential as a biomarker for disease severity.

While it was promising that inflammatory markers were reduced in both early and late hAEC treatment groups, it is unlikely that reduction of inflammation alone could wholly account for the reparative or protective effects of hAECs. Thus, we sought to ascertain whether hAECs were able to augment repair by promoting endogenous reparative responses. Previously, both mesenchymal stromal cells (MSCs) and MSC-conditioned media were reported to increase the number of BASCs in the terminal bronchioles of neonatal mice subjected to chronic hyperoxic insult [17]. We observed a similar finding in our current study, suggesting that hAECs may activate the bronchioalveolar junction stem cell niche as part of its pro-reparative actions in a manner akin to that observed in MSCs. However, the activation of AT2 cells, another progenitor cell in the distal lungs, did not change across treatment groups. One possibility could be that the AT2 cells were activated early on and this progenitor cell population had returned to a quiescent state by PND14. Another possibility which remains is that the mechanisms involved in AT2 cell and BASC proliferation and differentiation are different [16, 46–48] such that while hAECs are able to augment the BASC response they are unable to initiate the same cascade of events in AT2 cells.

Given the important physical relationship between the endothelial cells and stem/progenitor cells in lung repair, it was pertinent to investigate the relative impact of hAEC treatment on each of these cell types. When the BASC organoids were established stem/progenitor cells from each experimental group using healthy mouse lung endothelial cells as supporting stroma, we did not observe any differences in either the colony size or phenotype across all experimental groups. Our findings suggest that increased activation of the stem/progenitor cell compartment induced by hAEC treatment in vivo is likely due to an effect on the lung endothelial cells rather than the stem/progenitor cells themselves. As mentioned, endothelial cells have a significant influence on alveologenesis in vivo [23]. Likewise, stromal cells (mouse lung endothelial cells) are necessary to support BASC organoid cultures and can significantly influence BASC proliferation and differentiation in vitro [16]. By controlling for the contribution of the lung endothelial cells in our organoid cultures, we showed that the growth characteristics of the stem/progenitor cell population remained unchanged by hAEC treatment, thereby suggesting that the endothelial cells were the target cells of the beneficial effects of hAECs.

Vascular maldevelopment is another pathological hallmark of BPD where disrupted angiogenesis results in alveolar simplification and vascular muscularisation and ultimately leads to secondary pulmonary hypertension. Consistent with previous reports [49], we observed a reduction in the numbers of small pulmonary vessels (diameter < 50 μm) and associated alveolar simplification following antenatal inflammation and postnatal hyperoxia. This was mitigated in both early and late hAEC treatment groups. This is likely attributed to the secretion of pro-angiogenic factors including epidermal growth factor (EGF), angiogenin, vascular endothelial growth factor (VEGF), platelet-derived growth factor B (PDGFB) and angiogenin as reported previously by us and others [9, 50]. Vascular muscularisation begins in the peripheral pulmonary arteries, resulting in thicker and stiffer pulmonary artery walls with increased pulmonary vascular resistance. Thickening and muscularisation of pulmonary vessels can be detected as early as PND14 in our experimental model of BPD. These effects were diminished in both early and late hAEC treatment groups. When pulmonary vascular muscularisation progresses, it can cause secondary pulmonary hypertension, which happens to more than half of babies with severe BPD in some studies [32], thereby placing BPD survivors at continued cardiovascular risk [51].

It was with these long-term risks in mind that we assessed secondary pulmonary hypertension at 6 and 10 weeks, which represents mouse adolescence and early adulthood. Here, a decreased PAT/PET ratio detected on echocardiography is indicative of pulmonary hypertension [52]. We report that the experimental model of BPD used in our current study resulted in pulmonary hypertension as evident in decreased PAT/PET ratios in both adolescent and adult mice. While the PAT/PET ratio was improved in both early and late hAEC treatment groups this was only significant and wholly reversed in the early treatment group. Further to this, we observed evidence of right ventricular hypertrophy in both adolescence and early adulthood which was ameliorated by both treatment groups at week 6. However, this effect only persisted in the early hAEC treatment group. A similar study by Hansmann et al. [34] showed that the administration of MSC-conditioned media mitigated peripheral pulmonary artery muscularisation, pulmonary hypertension and right ventricular hypertrophy induced by postnatal hyperoxia (FiO_2_ = 0.75). However, it is important to note some key differences between our current study and that conducted by Hansmann et al. In their study, the newborn mice were exposed to postnatal hyperoxia, in the absence of an antenatal insult, for only 2 weeks before recovering in normoxia for 4 weeks prior to performing echocardiography. It therefore stands to reason that our current experimental model of BPD, which combines a longer period of postnatal hyperoxia with antenatal inflammation, may more closely replicate the clinical disease.

Long-term decline in lung function has been well documented in adult and adolescent survivors of BPD, including patients who only presented with mild respiratory distress [30, 53, 54]. We observed evidence of persistent increased airway responsiveness in the adolescent and the adult. Outcomes from the lung function tests indicated that intra-amniotic LPS and neonatal hyperoxia exposure led to increased lung resistance and decreased compliance. This pathology did not self-resolve and in fact persisted following a 2-week recovery period in normoxia. This is in keeping with a previous report by Regal et al. [55] which showed that changes in lung resistance and compliance persisted in mice exposed to 70% hyperoxia for 7 days followed by 14 days of recovery in normoxia. Notably the beneficial effects of hAEC treatment in this study were also persistent, particularly in the early hAEC treatment group, despite continued chronic hyperoxia exposure for 4 weeks following the intervention.

There are a number of potential reasons for the apparent longevity of a single dose of hAECs. Firstly, it is possible that the cells were administered within an ideal therapeutic window. Our study suggests that the ideal therapeutic window of BPD is closer to the initiation of injury. Conversely, the longevity of beneficial effects appears to be compromised when the hAECs are introduced after injury is established. This is similar to the therapeutic window for corticosteroid therapy for clinical BPD. Systemic postnatal corticosteroids used during the early stages of clinical management (≤ 7 days) can prevent the development of BPD [56], while postnatal steroids used later (> 7 days) only provide symptomatic relief of chronic lung diseases [57].

Another potential explanation is that hAEC administration may have epigenetic changes. Epigenetic modifications such as DNA methylation and histone modifications (e.g. acetylation, methylation, phosphorylation, ubiquitination) alter the chromatin conformation, resulting in activation or repression of gene expression [58]. hAEC treatment may result in epigenetic changes in targeted immune cells and/or local lung stem cells. For example, epigenetic changes associated with Wnt/β-catenin signalling may have influence inflammation or lung development. Wnt signalling is known to control leukocyte recruitment and macrophage activation [59]; it is essential to endothelial cell movement and proliferation in angiogenesis and vascular remodelling following injury [60]. Furthermore, key regulators of the Wnt signalling pathway are crucial to maintenance of epithelial stem cells [61–63]. These observations suggest that Wnt/β*-*catenin signalling is a point of intersection for inflammation, angiogenesis, alveologenesis and endogenous stem cell maintenance, which are key mechanisms shown in hAEC rescue of BPD lung injury.

In general, we have demonstrated that BPD mice had secondary pulmonary hypertension and decreased lung function, which was consistent with the long-term adverse effects observed clinically. Combined with our observations that neonatal hAEC treatment improved the long-term outcomes of experimental BPD in lung function and secondary cardiovascular changes, future clinical trials should include long-term follow-up.

There have been three clinical trials registered with ClinicalTrials.gov thus far, investigating the potential of MSCs (PNEUMOSTEM®) as a treatment for BPD, with only one trial completed to date (NCT01297205). This phase 1b trial recruited nine preterm infants born between 23 and 29 weeks, with a birth weight between 500 and 1250 g, and all infants required ventilation (> 12 breaths/min, > 25% oxygen) but were stable within 24 hours of enrolment. The other two clinical trials (NCT01828957, NCT02381366) had similar inclusion criteria. Critically, these criteria do not fulfil the NIH classifications for BPD, as not all babies requiring ventilation support will develop BPD. With this in mind, the phase 1b clinical trial we have commenced aims to assess the safety of hAEC administration to babies with established BPD, with the supportive information that late administration of hAECs improved functional outcomes after experimental BPD. However, given that our findings indicate greater benefits from earlier interventions, we anticipate that future hAEC trials will be designed with this in mind.

## Conclusions

We used a clinically relevant animal model that reproduces key pathological hallmarks of BPD, including respiratory and cardiovascular deficits that persist into adulthood. This study affords pre-clinical data that support investigations into the clinical utility of hAECs in BPD. Specifically, we report that hAECs mitigate lung injury in experimental BPD by restoring the tissue-to-airspace ratio, decreasing lung inflammation, modulating immune cell populations, activating the local stem cell niche and promoting vascular angiogenesis. Alveolar simplification and pruning of the pulmonary capillary bed was mitigated in both early (coinciding with the commencement of postnatal hyperoxia) and late (when lung injury is well underway) groups, while lung function was only improved in animals that received early hAEC intervention. Similarly, pulmonary hypertension and consequently right ventricular hypertrophy in adolescence and early adulthood were only mitigated in the early hAEC treatment groups. This finding suggests that hAEC therapy is more efficacious if given prophylactically. Future safety trials should therefore compare the efficacy of treatment in at-risk babies compared against babies with established disease, with long-term follow-up studies in order to evaluate their impact on cardiovascular and respiratory function later in life. Further studies to elucidate the long-term impact of repeated cell administration and the mechanisms of these legacy effects will aid and accelerate the development of hAEC-based cell therapies for BPD.

## Additional file

Additional file 1: Figure S1. Representative images of lung structure on PND7 by H&E staining, **Figure S2.** Representative images of pro-SPC and CC10 immunofluorescent staining on mouse lung tissues and **Figure S3.** BASCs and AT2 cells in in-vitro culture. (DOCX 1307 kb)

## Abbreviations

α-SMA: Alpha-smooth muscle actin
AT2: Type II alveolar epithelial cell
BASC: Bronchioalveolar stem cell
BPD: Bronchopulmonary dysplasia
CC10: Clara Cell 10 kD protein
Crs: Compliance
EGF: Epidermal growth factor
H&E: Haematoxylin and eosin
hAEC: Human amnion epithelial cell
IL: Interleukin
LIF: Leukemia inhibitory factor
LPS: Lipopolysaccharide
MCP: Monocyte chemoattractant protein
MIP: Macrophage inflammatory protein
MSC: Mesenchymal stem/stromal cell
PAT: Pulmonary artery acceleration time
PDGFB: Platelet-derived growth factor B
PET: Pulmonary artery ejection time
PND: Postnatal day
pro-SPC: Pro-surfactant protein C
PV loop: Pressure–volume loop
Rrs: Resistance
RVAWT: Right ventricle anterior wall thickness
TNF-α: Tumour necrosis factor alpha
VEGF: Vascular endothelial growth factor
vWF: von Willebrand factor
