# Supplementary material for: Human amnion cells reverse acute and chronic pulmonary damage in experimental neonatal lung injury
Source: Stem Cell Res Ther. 2017 Nov 10;8:257. doi: 10.1186/s13287-017-0689-9 (PMC5681809; doi:10.1186/s13287-017-0689-9)
Supplement: Additional file 1: Figure S1. — Representative images of lung structure on PND7 by H&E staining, Figure S2. Representative images of pro-SPC and CC10 immunofluorescent staining on mouse lung tissues and Figure S3. BASCs and AT2 cells in in-vitro culture. (DOCX 1307 kb) [file 13287_2017_689_MOESM1_ESM.docx]

Supplementary Materials for

**Human amnion cells reverse acute and chronic pulmonary damage in experimental neonatal lung injury**

Dandan Zhu, Jean Tan, Amina S. Maleken, Ruth Muljadi, Siow T. Chan, Sin N. Lau, Kirstin Elgass, Bryan Leaw, Joanne Mockler, Daniel Chambers, Kristen T. Leeman, Carla F. Kim, Euan M. Wallace, Rebecca Lim*

*Corresponding author Email: [Rebecca.lim@hudson.org.au](mailto:Rebecca.lim@hudson.org.au)

This File includes:

Figure. S1. Representative images of lung structure on PND7 as shown by H&E staining.

Figure. S2. Representative images of pro-SPC and CC10 immunofluorescent staining on mouse lung tissues.

Figure. S3. BASCs and AT2s *in vitro* culture.

**Supplementary Figures**


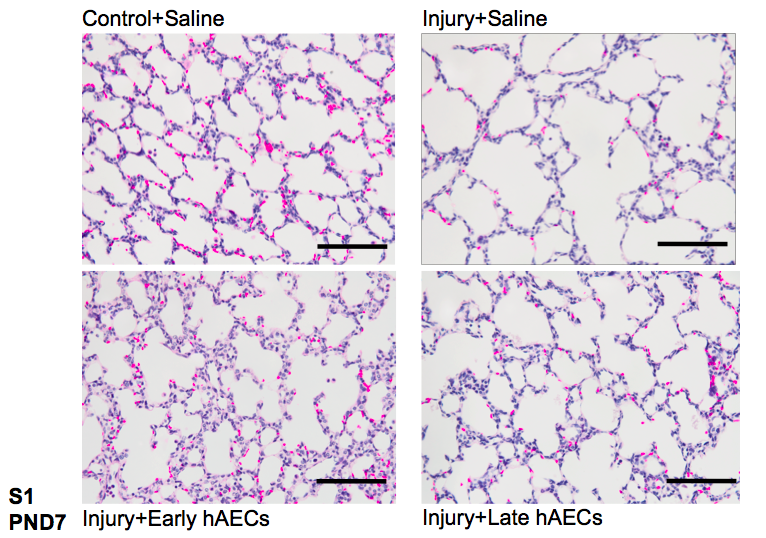


S1: Representative images of lung structure on PND7 as shown by H&E staining. Scale bar = 200μm


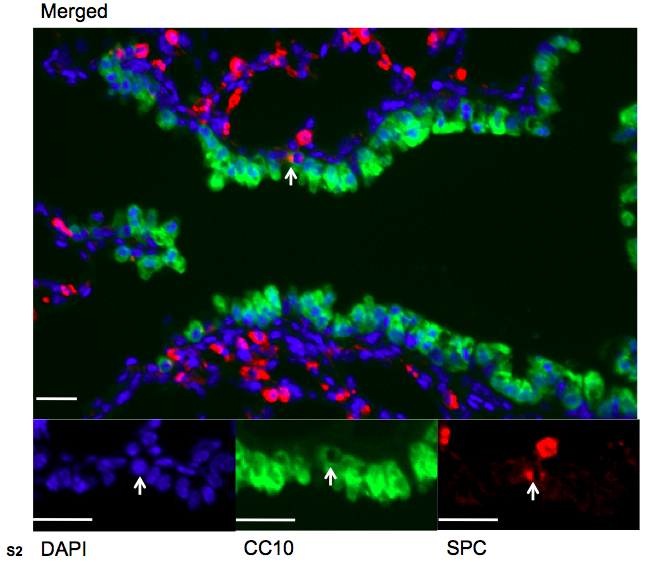


S2: Representative images of pro-SPC and CC10 immunofluorescent staining on mouse lung tissues. Arrows indicate BASCs (scale bars = 20μm).


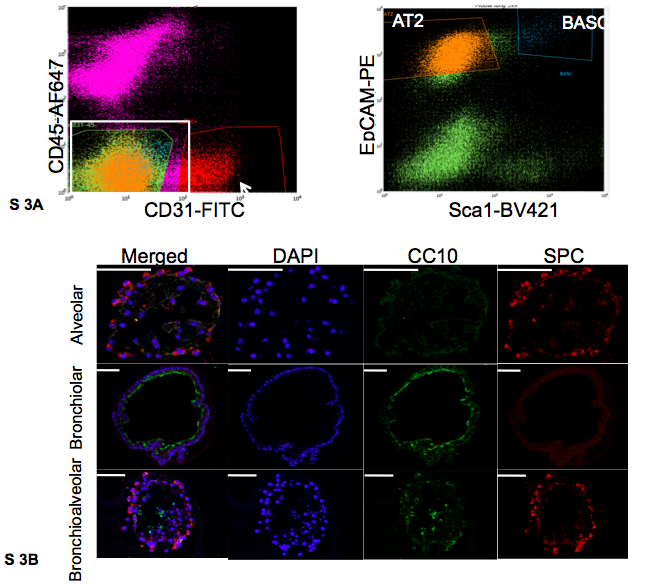


S3: BASCs and AT2s *in vitro* culture. (A) Representative FACS gates for BASCs and AT2s are provided where BASCs are CD45^neg^CD31^neg^Sca-1^pos^EpCAM^pos^, and AT2s are CD45^neg^CD31^neg^Sca-1^neg^EpCAM^pos^. (B) Representative images of pro-SPC and CC10 immunohistochemical staining on the alveolar, bronchiolar and bronchioalveolar organoids. Scale Bar=50μm.
